# Effect of the Substitution of the Mesityl Group with Other Bulky Substituents on the Luminescence Performance of [Pt(1,3-bis(4-Mesityl-pyridin-2-yl)-4,6-difluoro-benzene)Cl]

**DOI:** 10.3390/molecules30071498

**Published:** 2025-03-27

**Authors:** Giulia De Soricellis, Véronique Guerchais, Alessia Colombo, Claudia Dragonetti, Francesco Fagnani, Dominique Roberto, Daniele Marinotto

**Affiliations:** 1 Dipartimento di Chimica, Università degli Studi di Milano, UdR INSTM di Milano, Via C. Golgi 19, I-20133 Milan, Italy; giulia.desoricellis@unipv.it (G.D.S.); claudia.dragonetti@unimi.it (C.D.); dominique.roberto@unimi.it (D.R.); 2 Dipartimento di Chimica, Università di Pavia, Via Taramelli 12, I-27100 Pavia, Italy; 3 Univ. Rennes 1, CNRS, ISCR-UMR 6226, F-35000 Rennes, France; veronique.guerchais@univ-rennes.fr; 4 Istituto di Scienze e Tecnologie Chimiche (SCITEC) “Giulio Natta”, Consiglio Nazionale delle Ricerche (CNR), Via C. Golgi 19, I-20133 Milan, Italy; daniele.marinotto@cnr.it

**Keywords:** platinum(II) complexes, *N^C^N* ligand, luminescence

## Abstract

The synthesis and characterization of two new complexes, namely [Pt(bis(4-(4-(tert-butyl)phenyl)-pyridin-2-yl)-4,6-difluorobenzene)Cl] and [Pt(bis(4-(3,5-di-tert-butylphenyl)-pyridin-2-yl)-4,6-difluorobenzene)Cl], are reported. Both are highly luminescent in the blue region (Φlum = 0.89–0.95 at 478–480 nm), like the parent complex [Pt(1,3-bis(4-mesityl-pyridin-2-yl)-4,6-difluoro-benzene)Cl] in degassed diluted dichloromethane solution. An increase in concentration leads to the formation of bi-molecular emissive excited states, as evidenced by a growing structureless band that peaked at 690–697 nm. This formation is more facile for the complex with one tert-butyl group in para of the phenyl group. It appears that the introduction of two tert-butyls in positions 3 and 5 of the phenyl group hampers the neighboring of the monomeric species, although less efficiently than the introduction of methyls in positions 2 and 6.

## 1. Introduction

Square planar platinum(II) complexes are of prodigious interest in numerous fields of photonics and optoelectronics, such as nonlinear optics [1,2,3,4,5,6,7,8,9,10], artificial photosynthesis [11], photocatalysis [12,13,14], sensing [15,16,17,18,19,20,21,22,23,24,25,26,27,28,29], Organic Light Emitting Diodes (OLEDs) [30,31,32,33,34,35,36,37,38,39,40,41,42,43,44,45,46], bioimaging [47,48,49,50,51,52,53,54,55,56,57,58,59], and photodynamic therapy [60,61,62,63,64,65,66,67]. These complexes are characterized by a strong spin-orbit coupling due to the platinum center, which favors intersystem crossing and emission of light from triplet excited states [68,69]. Besides, their square planar geometry allows the formation of bimolecular states in the ground (dimers) and/or in the excited states (excimers) by means of Pt-Pt or ligand-ligand intermolecular interactions, a precious aspect because, for example, the parallel emissions from mono-molecular and bi-molecular excited states of platinum complexes allow the modulation of the color of luminescent devices [70].

In the last 20 years, a lot of work has been dedicated to platinum(II) chlorido complexes bearing a cyclometalated terdentate 1,3-bis(pyridin-2-yl)benzene ligand because they are among the brightest and most efficient emitters [71]. The parent complex [Pt(1,3-bis(pyridin-2-yl)benzene)Cl] is intensely luminescent in deoxygenated dichloromethane solution at room temperature (Φ_lum_ = 0.60 and τ = 7.2 μs) [72], in striking contrast to [Pt(terpyridine)Cl]^+^, which is essentially non-emissive because it has low-lying d-d states that allow a thermally activated non-radiative decay pathway for the MLCT state [73]. In the complex [Pt(1,3-bis(pyridin-2-yl)benzene)Cl], the presence of the cyclometallating carbon causes an increase in the ligand-field strength and, thus, in the energy of the d-d state, removing this decay pathway [71]. Besides, the rigidity of the tridentate ligand has a beneficial effect on respect to bidentate ligands. In fact, the complex [Pt(ppy)(Hppy)Cl] (ppyH is phenylpyridine) also has a coordination sphere with one cyclometallated benzene ring, two pyridine rings, and one chloride ligand; however, the distortion of the bidentate ligands leads to non-radiative decay at room temperature: the luminescence lifetime is very short at room temperature (641 ns) and increases a lot at 77 K (11.2 μs) [74]. In contrast, the luminescence lifetime of [Pt(1,3-bis(pyridin-2-yl)benzene)Cl] is the same at 77K and at room temperature, showing that the greater rigidity of the tridentate ligand prevents the distortion [71]. Besides, the *N^N^C*-coordinated [Pt(phenylbipyridine)Cl] isomeric complex has a luminescence quantum yield an order of magnitude lower, although it has a tridentate ligand because the Pt–C bond in [Pt(1,3-bis(pyridin-2-yl)benzene)Cl] is shorter than in [Pt(phenylbipyridine)Cl] leading to a higher ligand-field strength [71].

Remarkably, the absorption and emission maxima of [Pt(1,3-bis(pyridin-2-yl)benzene)Cl] derivatives are influenced by the introduction of electron-donor and/or electron-acceptor substituents on the cyclometallated benzene and on the pyridine rings, offering a versatile method of color tuning. Thus, time-dependent density functional theory (TD-DFT) calculations showed that the frontier orbitals are on different portions of the complex, such that their energies can be modified almost independently [75]. In this class of compounds in which the triplet state at the lowest energy has mainly HOMO→LUMO character, the benzene ring gives the main contribution to the HOMO, whereas the pyridine rings control the LUMO. Therefore, the presence of electron-donor substituents in the benzene ring (which raises the HOMO energy) and electron-acceptor substituents in the pyridine rings (which lowers the LUMO energy, especially when in position *para* with respect to the N atoms) will lead to a red-shift in the emission, while electron-acceptor substituents in the benzene ring and electron-donor substituents in the pyridine rings will cause a blue-shift. The experimentally observed tendencies follow this design well [48,71,76].

Interestingly, these platinum(II) chlorido complexes with a cyclometalated 1,3-bis(pyridin-2-yl)-benzene ligand show two phosphorescence bands situated in the bluish-green (monomeric emission) and red (excimer/aggregate emission) regions of the visible spectrum, which contributions to the global emission can be chosen by the amount of the platinum complex in the host matrix used for the preparation of the OLED’s blend emissive layer. This represents a precious tool for tailoring the emission color of OLEDs [32].

**Scheme 1:**
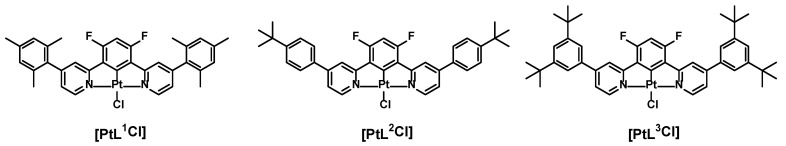
Chemical structures of the investigated Pt(II) complexes.

However, today, the preparation of efficient blue phosphorescent OLEDs remains a challenge [77,78,79]. The idea of filling this gap prompted us to investigate the effect of the introduction of a bulky substituent on position 4 of the pyridine rings of the complex with a fluorinated cycloplatinated 1,3-bis(pyridin-2-yl)-4,6-difluoro-benzene ligand, for which the presence of the fluorine atoms allows a blue monomeric emission. The idea was that the knowledge of such an effect would be of particular interest because excimers or aggregates formation, and, therefore, the phosphorescence properties, could be controlled, achieving the proper spatial arrangement of bulky functional groups, which could decrease Pt-Pt interactions and/or π−π stacking and the concomitant red emission. Promising results were reached recently with the preparation of a chlorido platinum(II) complex bearing a well-designed new *N^C^N*-cyclometalating ligand, namely 1,3-bis(4-mesityl-pyridin-2-yl)-4,6-difluoro-benzene (Scheme 1, **[PtL^1^Cl]**) [44]. Thus, the introduction of the bulky mesityl substituent on the pyridine rings turned out to be a facile strategy to increase the Pt···Pt distance, reaching a value (8.59 Å) much longer than that observed for previously reported chlorido *N^C^N*-platinum(II) complexes. The **[PtL^1^Cl]**) complex, which exhibits intense blue phosphorescence in dilute dichloromethane solution (471 nm, Φ_lum_ = 0.97), was used to fabricate a blue OLED with an excellent performance when compared to that reached with blue OLEDs based on other platinum complexes [44]. Clearly, the introduction of bulky substituents in *N^C^N* Pt(II) complexes represents a springboard for the fabrication of efficient blue OLEDs. For this reason, we designed, prepared, and thoroughly characterized in solution two novel complexes, namely **[PtL^2^Cl]** and **[PtL^3^Cl]**, with one or two *tert*-butyl groups on the phenyl group in position 4 of the pyridine rings (Scheme 1).

## 2. Results and Discussion

### 2.1. Preparation of the Complexes

**Scheme 2:**
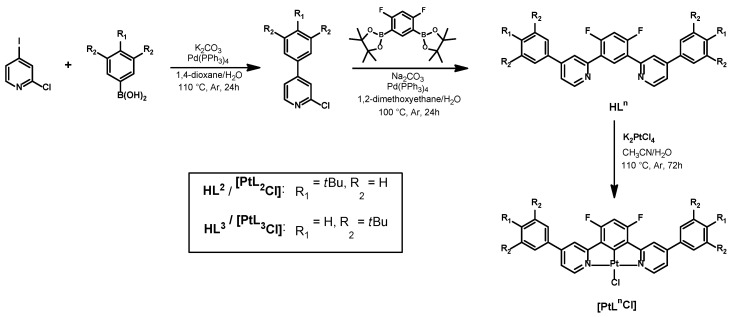
Synthesis of complexes **[PtL^2^Cl]** and **[PtL^3^Cl]**.

The new pro-ligands 1,3-bis(4-(4-(*tert*-butyl)phenyl)pyridin-2-yl)-4,6-difluoro-benzene (HL^2^) and 1,3-bis(4-((3,5-di-*tert*-butylphenyl))-pyridin-2-yl)-4,6-difluoro-benzene (HL^3^) were prepared by cross-coupling the pinacol ester of 1,3-difluoro-4,6-diboronic acid [44] with the suitable 2-chloro-4-Aryl-pyridine (Scheme 2). The reaction of these pro-ligands with K_2_PtCl_4_ led to the desired complexes in 41% yield ([PtL^2^Cl]) and 85% yield ([PtL^3^Cl]). The complexes were fully characterized by NMR (^1^H, ^13^C, and ^19^F) spectroscopy, mass spectrometry (HRMS), and elemental analysis. Full details of the synthesis and characterization are provided in the Section 3 “Materials and Methods” and in the Supporting Information.

### 2.2. Photophysical Properties

**Figure 1:**
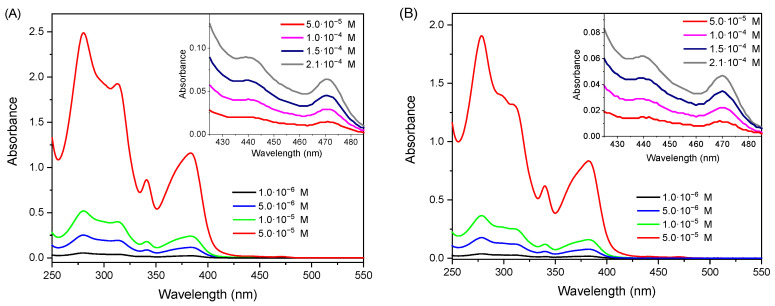
Absorption spectra of **[PtL^2^Cl]** (**A**) and **[PtL^3^Cl]** (**B**) at different concentrations in CH_2_Cl_2_ at 298 K. The weak bands at longer wavelengths are shown on an expanded scale for clarity.

The absorption spectra of compounds **[PtL^2^Cl]** and **[PtL^3^Cl]** at various concentrations (1·10^−6^–2·10^−4^ M) in CH_2_Cl_2_ are shown in Figure 1. There are bands of high intensity in the range 260–320 nm, attributed to intraligand ^1^π–π* transitions of the *N^C^N* cyclometalating ligand, and bands of lower intensity at 340–420 nm, caused by charge-transfer transitions concerning the cyclometalating ligand and the metal, as for other *N^C^N* platinum(II) complexes [39,40,41,42,43,44,70,71,72,73,80]. A weak absorption band at 470 nm is also present in the absorption spectra of both **[PtL^2^Cl] (**ε = 310 L mol^−1^ cm^−1^) and **[PtL^3^Cl]** (ε = 225 L mol^−1^ cm^−1^), as shown in the insets of Figure 1, due to the weak transition from the singlet ground state to the lowest triplet state, facilitated by the presence of the platinum center [80].

**Figure 2:**
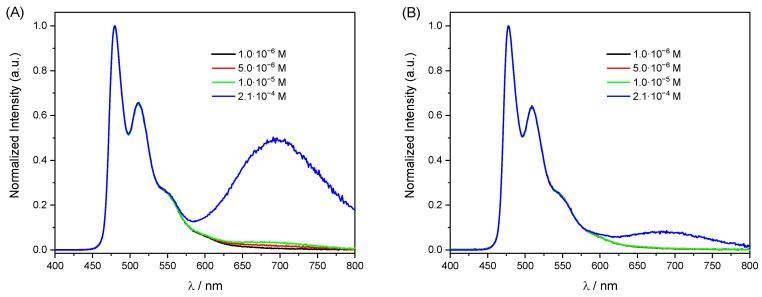
Normalized emission spectra of **[PtL^2^Cl]** (**A**) and **[PtL^3^Cl]** (**B**) after excitation at 380 nm, at different concentrations in deaerated CH_2_Cl_2_ solution at 298 K.

The normalized emission spectra at room temperature of **[PtL^2^Cl]** and **[PtL^3^Cl]** in degassed dichloromethane at various concentrations are shown in Figure 2. Upon excitation at 380 nm in dilute solution (1∙10^−6^ M), the emission spectrum of both complexes is characterized by a vibrationally structured band with a high-energy emission maximum at 480 nm (for **[PtL^2^Cl]**) and 478 nm (for **[PtL^3^Cl]**), slightly red-shifted with respect to that of **[PtL^1^Cl]** (471 nm) [44]. This band can be attributed to the electronic T_1_ → S_0_ transition of the monomeric complex [80].

**Figure 3:**
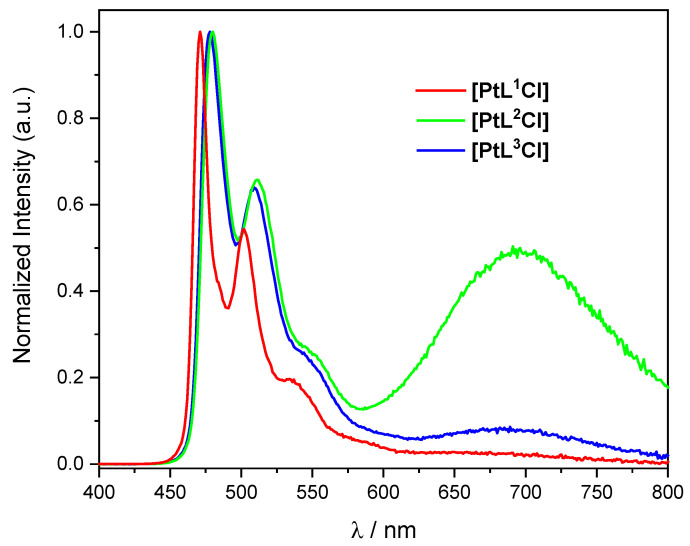
Normalized emission spectra of **[PtL^1^Cl]**, **[PtL^2^Cl]**, and **[PtL^3^Cl]** after excitation at 380 nm, in concentrated degassed CH_2_Cl_2_ solution (2·10^−4^ M) at 298 K.

As expected from the behavior of other *N^C^N* platinum complexes [32,39,40,41,42,43], when the concentration of **[PtL^2^Cl]** and **[PtL^3^Cl]** is increased up to 2·10^−4^ M, the emission spectra show a growing structureless band peaked at 690–697 nm, in addition to the vibrationally structured band. This structureless band at lower energy, due to bi-molecular emissive excited states (excimers and aggregates) of the platinum (II) complex formed by means of Pt-Pt or π−π ligand-ligand intermolecular interactions [32], is much more intense for **[PtL^2^Cl]** than for **[PtL^3^Cl]** (see Figure 2A,B). The formation of aggregates was confirmed by means of excitation spectra (see Electronic Supporting Information). Figure 3 shows the normalized emission spectrum of both complexes with that of **[PtL^1^Cl]** in concentrated degassed CH_2_Cl_2_ solution (2·10^−4^ M). It puts in evidence that the low energy band intensity follows the order **[PtL^2^Cl]** > > **[PtL^3^Cl]** > **[PtL^1^Cl],** highlighting that the presence of the *tert*-butyl group in *para* of the phenyl group is much less efficient in hampering the formation of dimeric species. The introduction of two *tert*-butyl groups (**[PtL^3^Cl]**) leads to a clear decrease in the intensity of the structureless band, in agreement with an increase in the steric hindrance. It appears that the presence of a mesityl group in *para* of the pyridine rings is even more efficient in hampering the approach of the monomeric species. In the complex **[PtL^1^Cl]**, there is a torsion angle of ca 62° between the mesityl groups and the pyridines, as shown from the X-ray structure, which hinders the neighboring of the metal atoms and, therefore, the Pt···Pt interactions; the shortest Pt···Pt distance is 8.59 Å much longer than that observed for the related platinum(II) chlorido complex with a cyclometalated 5-mesityl-1,3-bis(pyridin-2-yl)-benzene ligand (4.4 Å) [44]. Unfortunately, various attempts to obtain crystals of **[PtL^2^Cl]** and **[PtL^3^Cl]** suitable for X-ray determination failed. In any case, the decrease in the intensity of the structureless band at low energy on going from **[PtL^2^Cl]** to **[PtL^3^Cl]** is expected upon the introduction of a second *tert*-butyl group on the phenyl ring. Besides, our results put in evidence the importance of having substituents in the alpha of the carbon linked to the pyridine to better hamper the neighboring of the monomeric species.

In degassed diluted CH_2_Cl_2_ solution at room temperature, it is known that **[PtL^1^Cl]** is a bright Pt(II) emitter with a luminescence quantum yield (Φ_lum_) of 0.97 [44]. The two new complexes are also characterized by excellent absolute quantum yields, 0.89 and 0.95 for **[PtL^2^Cl]** and **[PtL^3^Cl]**, respectively, as determined with an integrating sphere (Table 1). When the solution of the complexes is air-equilibrated, the Φ_lum_ decreases to 0.26 and 0.21 due to oxygen quenching, as expected from the behavior of **[PtL^1^Cl]** and similar platinum compounds [44]. Thus, it is worth pointing out that an efficient production of singlet oxygen can be anticipated for these complexes, an interesting aspect of photodynamic therapy [65].

It is known that an increase in the concentration usually leads to a strong decrease in the luminescence quantum yield of *N^C^N* platinum(II) due to the formation of aggregates and excimers species [71]. As expected, an increase in the concentration of the degassed dichloromethane solution causes a drop in the quantum yield, which, however, remains very high (Φ_lum_ = 0.57 and 0.54 at 2·10^−4^ M, for **[PtL^2^Cl]** and **[PtL^3^Cl]**, respectively) like previously observed for **[PtL^1^Cl]** (Φ_lum_ = 0.62 [44]). Interestingly, these complexes with a bulky phenyl group on the pyridine rings are suitable for keeping excellent quantum yields even in concentrated solutions.

Excited state decay measurements of the new complexes in degassed CH_2_Cl_2_ solutions at different concentrations were performed, exciting at 374 nm at the emission wavelength of 480 and 478 nm for **[PtL^2^Cl]** (ESI, Table S2 and Figures S4–S7) and **[PtL^3^Cl]** (ESI, Table S4 and Figures S11–S14). The longest lifetime, 4.06–4.09 μs, is observed at the lowest concentration (1·10^−6^ M, ESI). An increase in the concentration leads to a decrease in the lifetime (Table 1), as expected for the formation of bimolecular states with a diminution of both lifetime and quantum yield. Figures S3 and S10 in the ESI report the excitation spectra in solution at low (1·10^−6^ M) and high (2·10^−4^ M) concentrations for **[PtL^2^Cl]** and **[PtL^3^Cl]**, showing the variations of the spectra due to the presence of aggregate species, with a new broadband clearly visible in the region 500–650 nm.

It is worth pointing out that the drop of the lifetime on going from a concentration of 1·10^−6^ M to 2·10^−4^ M is much larger for **[PtL^2^Cl]** than for **[PtL^3^Cl]** (Table 1). This observation would confirm a more difficult formation of excimers/aggregates in the case of **[PtL^3^Cl]**, reasonably due to the steric hindrance of the two *tert*-butyl groups on the phenyl linked to the pyridine rings. Such an effect was previously observed for **[PtL^1^Cl]** with the pyridines bearing a mesityl group, where the τ value at 2·10^−4^ M was half that measured at 5·10^−6^ M (Table 1) [44].

The slightly higher Φ_lum_ in the dilute solution of **[PtL^3^Cl]** with respect to **[PtL^2^Cl]** is reflected by a lower non-radiative rate constant (k_nr_) for the former (Table 1).

## 3. Materials and Methods

All the reagents and the solvents (dioxane, 1,2-dimethoxyethane, acetonitrile) were used as received from the supplier. Anhydrous toluene was obtained via distillation under argon in the presence of benzophenone. The purifications were performed through column chromatography on silica gel (063–0.200 mm, Merck, Darmstadt, Germany). Grace RevelerisTM with PuriflashTM 40 µm flash cartridges (Buchi, Uster, Switzerland) was used to perform flash chromatography.

The NMR characterizations were obtained by recording on Bruker AV III 300 MHz or AV III 400 MHz spectrometers (Bruker, Billerica, MA, USA). The chemical shifts of ^1^H, ^19^F, and 13C NMR spectra are reported in parts per million (ppm), and the coupling constants are measured in Hertz (Hz). The multiplicities of signals are listed as singlet (s), d (doublet), t (triplet), quartet (q), and multiplet (m).

The molecules were analyzed through mass spectrometry at the Centre Régional de Mesures Physiques de l’Ouest, University of Rennes 1, on an LC-MS Agilent 6510 (Agilent, Santa Clara, CA, USA), a Brucker MaXis 4G, or a Thermo Fisher Q-Exactive (Thermo Fisher, Waltham, MA, USA) using ESI and ASAP techniques. Elemental analyses were performed by the Department of Chemistry of the University of Milan.

Electronic absorption spectra in solution were obtained with a UV-3600i Plus UV-VIS-NIR spectrophotometer (Shimadzu Italia S.r.l., Milan, Italy). Luminescence measurements were carried out in CH_2_Cl_2_ solution after the Freeze-Pump-Thaw (FPT) procedure to remove dissolved oxygen. Absolute photoluminescence quantum yield, Φ, was measured using a C11347 Quantaurus Hamamatsu Photonics K.K spectrometer (See ESI).

### 3.1. Procedure for the Synthesis of ***[PtL^2^Cl]***

#### 3.1.1. Synthesis of HL^2^

**4-(4-(*tert*-butyl)phenyl)-2-chloropyridine (1):**

A round-bottom flask was charged with 4-*tert*-butylbenzeneboronic acid (1.49 g, 8.4 mmol), 2-chloro-4-iodopyridine (1.00 g, 4.2 mmol), and K_2_CO_3_ (1.73 g, 12.5 mmol) under an argon atmosphere. The reagents were solubilized in a 2:1 mixture of 1,4-dioxane and water. An argon needle was placed in the reaction mixture for 30 min to eliminate oxygen traces. Pd(PPh_3_)_4_ (0.24 g, 0.2 mmol) was then added, and the reaction vessel was sealed. The mixture was heated under reflux for 72 h and then cooled to room temperature. The organic phase was extracted with toluene, washed three times with 30 mL of water and 20 mL of brine, dried over anhydrous MgSO_4_, and concentrated in vacuo. Purification via silica gel chromatography (cyclohexane:AcOEt, 20:1, v/v) yielded a colorless oil (547 mg, 53%). ^1^H NMR (300 MHz, CDCl_3_, δ): 8.43 (d, J = 5.2 Hz, 1H), 7.48–7.64 (m, 5H), 7.44 (dd, J = 5.2, 1.6 Hz, 1H), 1.39 (s, 9H).^13^C NMR (75.48 MHz, CDCl_3_, δ): 153.2, 152.3, 151.4, 150.0, 133.8, 126.7, 126.2, 121.9, 120.4, 34.9, 31.2. HRMS (ESI+): (M + Na)^+^ calcd for C_15_H_16_N^35^ClNa, 268.0869; found: 268.0863.

**2,2′-(4,6-difluoro-1,3-phenylene)bis(4-(4-(tert-butyl)phenyl)pyridine) (HL2):**

Compound 1 (0.55 g, 2.2 mmol), the pinacol ester of benzene-1,3-difluoro-4,6-diboronic acid (0.36 g, 0.99 mmol), an aqueous Na_2_CO_3_ solution (1 M, 13 mL) and Pd(PPh_3_)_4_ (0.19 g, 0.17 mmol) were placed in a Schlenk tube and dissolved in 13 mL of DME. The system was degassed under an argon flow for 10 min before sealing the vessel. The reaction was heated at 100 °C overnight. After cooling, the solvent was evaporated in vacuo, and the oily residue was dissolved in dichloromethane (30 mL) and water (20 mL). The organic layer was collected, dried over anhydrous MgSO_4_, and concentrated. The crude product was purified by silica gel chromatography (cyclohexane/AcOEt, 16:1) to afford a colorless oil (501 mg, 95%). ^1^H NMR (300 MHz, CDCl_3_, δ): 8.77 (d, J = 5.1 Hz, 2H), 8.70 (t, J = 9 Hz, 1H), 8.01 (s, 2H), 7.68 (d, J = 8.5 Hz, 4H), 7.56 (d, J = 8.5 Hz, 4H), 7.51 (dd, J = 5.2, 1.7 Hz, 2H), 7.10 (t, J = 10.7 Hz, 1H), 1.41 (s, 18H).^13^C NMR (75.48 MHz, CDCl_3_, δ): 153.1, 152.5, 150.2, 148.9, 135.4, 134.0, 126.9, 126.2, 122.1, 120.6, 105.1, 34.9, 34.7, 34.4, 31.5, 31.3, 30.2, 29.7.^1^⁹F NMR (282.36 MHz, CDCl_3_, δ): −112.5 (s, 2F). HRMS (ASAP): (M + H)⁺ calcd for C_36_H_34_N_2_F_2_ 533.2763; found: 533.2764.

#### 3.1.2. Synthesis of **[PtL^2^Cl]**

A Schlenk tube was charged with HL^2^ (0.10 g, 0.19 mmol) and dissolved in 9 mL of acetonitrile. Separately, K_2_PtCl_4_ (0.16 g, 0.38 mmol) was dissolved in 1 mL of water and added to the reaction vessel using a Pasteur pipette. The system was degassed for 40 min under an argon stream. The reaction mixture was then sealed and heated at 110 °C for 3 days. The resulting yellow suspension was cooled to room temperature and filtered through a nylon membrane filter. The obtained yellow solid was washed with water and diethyl ether and then dried to afford 77 mg (41%) of the product. ^1^H NMR (300 MHz, CDCl_3_, δ): 9.27 (d, J = 5.9 Hz, 3J(^1^⁹⁵Pt) = 39.3 Hz, 2H), 8.10 (s, 2H), 7.69 (d, J = 8.3 Hz, 4H), 7.58 (d, J = 8.3 Hz, 4H), 7.46 (dd, J = 5.9, 1.9 Hz, 2H), 6.73 (t, J = 11.2 Hz, 1H), 1.41 (s, 18H). ^13^C NMR (75.48 MHz, CDCl_3_, δ): 153.7, 151.7, 133.6, 126.7, 126.3, 120.3, 34.7, 31.1, 27.1. ^1^⁹F NMR (282 MHz, CDCl_3_, δ): –109.00 (s, 2F). HRMS (ASAP): (M + H_2_O)⁺ calcd for C_36_H_35_N_2_OF_2_^1^⁹⁵Pt, 744.2360; found: 744.2367. Elemental Analysis (calcd for C_36_H_33_ClF_2_N_2_Pt): C, 56.73; H, 4.36; N, 3.68; found: C, 56.77; H, 4.35; N, 3.70.

### 3.2. Procedure for the Synthesis of ***[PtL^3^Cl]***

#### 3.2.1. Synthesis of HL^3^

**2-chloro-4-(3,5-di-tert-butylphenyl)pyridine (2):**

A round-bottom flask was charged with (3,5-di-tert-butylphenyl)boronic acid (0.88 g, 3.8 mmol), 2-chloro-4-iodopyridine (0.50 g, 2.1 mmol), and K_2_CO_3_ (0.87 g, 6.3 mmol) under an argon atmosphere. The reagents were dissolved in a 2:1 mixture of 1,4-dioxane and water. The reaction mixture was degassed by bubbling argon for 30 min. Pd(PPh_3_)_4_ (0.24 g, 0.2 mmol) was then added and the vessel was sealed. The reaction was heated at reflux for 72 h, then allowed to cool to room temperature. The organic phase was extracted with toluene, washed three times with 30 mL of water and 20 mL of brine, dried over anhydrous MgSO_4_, and concentrated in vacuo. Purification via silica gel chromatography (cyclohexane:AcOEt, 20:1, *v*/*v*) afforded a colorless oil (847 mg, 67%). ^1^H NMR (300 MHz, CDCl_3_, δ): 8.47 (d, J = 5.2 Hz, 1H), 7.55–7.60 (m, 2H), 7.43–7.48 (m, 3H), 1.42 (s, 18H).

**2,2′-(4,6-difluoro-1,3-phenylene)bis(4-(3,5-di-tert-butylphenyl)pyridine) (HL3):**

Compound 2 (0.42 g, 1.4 mmol), the pinacol ester of benzene-1,3-difluoro-4,6-diboronic acid (0.23 g, 0.6 mmol), an aqueous Na_2_CO_3_ solution (1 M, 8 mL), and Pd(PPh_3_)_4_ (0.12 g, 0.1 mmol) were placed in a Schlenk tube and dissolved in 8 mL of DME. The system was degassed under an argon flow for 10 min and then sealed. The reaction mixture was heated at 100 °C overnight. After cooling, the solvent was evaporated in vacuo, and the residue was dissolved in dichloromethane (30 mL) and water (20 mL). The organic phase was collected, dried over anhydrous MgSO_4_, filtered, and concentrated. Purification via silica gel chromatography (cyclohexane/AcOEt, 16:1, *v*/*v*) yielded a colorless oil (438 mg, 68%). ^1^H NMR (300 MHz, CDCl_3_, δ): 8.79 (d, J = 5.1 Hz, 2H), 8.70 (t, J = 8.9 Hz, 1H), 8.0 (s, 2H), 7.47–7.62 (m, 8H), 7.11 (t, J = 10.6 Hz, 1H), 1.43 (s, 36H). ^13^C NMR (75.48 MHz, CDCl_3_, δ): 162.5, 162.1, 158.9, 158.8, 153.0, 151.7, 150.4, 150.0, 137.8, 133.8, 124.7, 123.3, 122.7, 121.6, 121.1, 105.4, 105.1, 104.7, 35.0, 31.5, 30.2, 29.7. ^1^⁹F NMR (282.36 MHz, CDCl_3_, δ): −112.92 (s, 2F). HRMS (ESI⁺): (M + H)⁺ calcd for C_44_H_51_N_2_F_2_, 645.4015; found: 645.4008.

#### 3.2.2. Synthesis of **[PtL^3^Cl]**

A Schlenk tube was charged with HL^2^ (0.17 g, 0.27 mmol) and dissolved in 11.7 mL of acetonitrile. Separately, K_2_PtCl_4_ (0.22 g, 0.54 mmol) was dissolved in 1.3 mL water and added to the reaction vessel using a Pasteur pipette. The system was degassed by bubbling argon directly into the solvent for 40 min. The reaction was then sealed and heated at 110 °C for 3 days until a yellow suspension formed. After cooling to room temperature, the mixture was filtered through a nylon membrane filter, yielding a yellow solid. The solid was washed with water, methanol, and diethyl ether and then dried to afford 148 mg (85%) of the product. ^1^H NMR (300 MHz, CDCl_3_, δ): 9.34 (d, J = 6.0 Hz, 3J(^1^⁹⁵Pt) = 39.4 Hz, 2H), 8.13 (s, 2H), 7.63 (s, 2H), 7.45–7.59 (m, 6H), 6.74 (t, J = 11.2 Hz, 1H), 1.43 (s, 36H). ^13^C NMR (75.48 MHz, CDCl_3_, δ): 164.3, 153.3, 152.0, 151.7, 136.6, 124.5, 121.5, 121.1, 35.2, 31.4. ^1^⁹F NMR (282 MHz, CDCl_3_, δ): −108.48 (s, 2F). HRMS (ESI⁺): (M + Na)⁺ calcd for C_44_H_49_N_2_F_2_^3^⁵Cl Na^1^⁹⁵Pt, 896.3092; found: 896.3085. Elemental Analysis (calcd for C_44_H_49_ClF_2_N_2_Pt): C, 60.44; H, 5.65; N, 3.20; found: C, 60.48; H, 5.66; N, 3.23.

## 4. Conclusions

In conclusion, two novel complexes, namely [Pt(bis(4-(4-(*tert*-butyl)phenyl)-pyridin-2-yl)-4,6-difluorobenzene)Cl] and [Pt(bis(4-(3,5-di-*tert*-butylphenyl)-pyridin-2-yl)-4,6-difluorobenzene)Cl] were easily prepared and thoroughly characterized. In degassed diluted dichloromethane solution, both are highly luminescent (Φlum = 0.89–0.95) in the blue region (478–480 nm) with a lifetime similar to that usually observed for *N^C^N* platinum(II) complexes (ca 4 µs). Like for the parent complex [Pt(1,3-bis(4-mesityl-pyridin-2-yl)-4,6-difluoro-benzene)Cl], excellent luminescent quantum yields are obtained even in concentrated solution (Φlum = 0.54–0.57).

An important aspect that this work puts in evidence is the effect on the formation of excimers/aggregates of the substituents on the phenyl group linked in the *para* position of the pyridines. The steric effect caused by the presence of three methyls in positions 2, 4, and 6 of the phenyl (i.e., the mesityl group) seems the best to hamper the neighboring of monomeric complexes, as evidenced by the lower intensity of the low energy band characteristic of excimers/aggregates in concentrated solutions. The presence of two *tert*-butyls in positions 3 and 5 of the phenyl group appears slightly less efficient but better than one *tert*-butyl in the *para* of the phenyl group. Clearly, it is imperative to have substituents in the alpha of the carbon linked in the *para* position of the pyridine to hamper better the neighboring of the monomeric species, which would allow Pt-Pt and π−π interactions with concomitant red emission. This observation represents an important guideline for the design of *N^C^N* platinum complexes as efficient emitters for blue OLEDs.

## Figures and Tables

**Table 1:** Photophysical parameters ^a^ of **[PtL^2^Cl]** and **[PtL^3^Cl]** in comparison with that of **[PtL^1^Cl]** [44].

	λ_max,abs_ (S_0_→T_1_)/nm [ε/M^−1^cm^−1^]	λ_max,em_/nm ^b^ Monomer [excimer/Aggregate] ^c^	Φ_lum_ ^d^ [Aerated]	τ/µs	k_r_^e^/s^−1^	k_nr_^e^/s^−1^
**[PtL^1^Cl]** (5·10^−6^M)	467 [120]	471 [680]	0.97 [0.18]	4.77	2.0·10^5^	6.3·10^3^
**[PtL^1^Cl]** (2·10^−4^M)			0.62 [0.12]	2.41	2.6·10^5^	1.6·10^5^
**[PtL^2^Cl]** (5·10^−6^M)	470 [310]	480 [697]	0.89 [0.26]	3.91	2.3·10^5^	2.8·10^4^
**[PtL^2^Cl]** (2·10^−4^M)			0.57 [0.23]	1.17	4.9·10^5^	3.7·10^5^
**[PtL^3^Cl]** (5·10^−6^M)	470 [225]	478 [690]	0.95 [0.21]	3.96	2.4·10^5^	1.3·10^4^
**[PtL^3^Cl]** (2·10^−4^M)			0.54 [0.17]	2.11	2.6·10^5^	2.2·10^5^

^a^ at 298 K in degassed CH_2_Cl_2_. ^b^ Excitation at 330 nm. ^c^ Excimer/aggregate at 2·10^−4^ M. ^d^ Absolute Φ_lum_ measured with an integrating sphere. ^e^ Radiative and nonradiative rate constants are calculated from the quantum yields and emission decay lifetimes, according to Φ_lum_ = k_r_·τ_em_ = k_r_/(k_r_ + k_nr_).

## Data Availability

The full characterization of the two complexes (^1^H, ^13^C, and ^19^F NMR spectra, HRMS, elemental analysis, and photophysical characterization) have been included as part of the Supplementary Information.

## Supplementary Materials

The following supporting information can be downloaded at: https://www.mdpi.com/article/10.3390/molecules30071498/s1. ^1^H, ^13^C and ^19^F NMR spectra of intermediates, ligands and Pt(II) complexes; Table S1: Molar extinction coefficients for [PtL^2^Cl]; Figures S1 and S2: Absorbance vs Concentration for [PtL^2^Cl]; Figure S3: Excitation spectra for [PtL^2^Cl]; Table S2: Lifetimes values for [PtL^2^Cl]; Figures S4–S7: Lifetime measurements for [PtL^2^Cl]; Table S3: Molar extinction coefficients for [PtL^3^Cl]; Figures S8 and S9: Absorbance vs Concentration for [PtL^3^Cl]; Figure S10: Excitation spectra for [PtL^3^Cl]; Table S4: Lifetimes values for [PtL^3^Cl]; Figures S11–S14: Lifetime measurements for [PtL^3^Cl]; [81].

## References

[1] Deplano P., Pilia L., Espa D., Mercuri M.L., Serpe A. (2010). Square-planar d^8^ metal mixed-ligand dithiolene complexes as second order nonlinear optical chromophores: Structure/property relationship. Coord. Chem. Rev..

[2] Colombo A., Dragonetti C., Marinotto D., Righetto S., Roberto D., Tavazzi S., Escadeillas M., Guerchais V., Le Bozec H., Boucekkine A. (2013). Cyclometallated 4-styryl-2-phenylpyridine Pt(II) acetylacetonate complexes as second-order NLO building blocks for SHG active polymeric films. Organometallics.

[3] Espa D., Pilia L., Marchiò L., Mercuri M.L., Serpe A., Barsella A., Fort A., Dalgleish S.J., Robertson N., Deplano P. (2011). Redox-switchable chromophores based on metal (Ni, Pd, Pt) mixed-ligand dithiolene complexes showing molecular second-order nonlinear-optical activity. Inorg. Chem..

[4] Espa D., Pilia L., Marchiò L., Pizzotti M., Robertson N., Tessore F., Mercuri M.L., Serpe A., Deplano P. (2012). Electrochromic second-order NLO chromophores based on M^II^ (M = Ni, Pd, Pt) complexes with diselenolato–dithione (donor–acceptor) ligands. Dalton Trans..

[5] Espa D., Pilia L., Makedonas C., Marchiò L., Mercuri M.L., Serpe A., Barsella A., Fort A., Mitsopoulou C.A., Deplano P. (2014). Role of the acceptor in tuning the properties of metal [M(II) = Ni, Pd, Pt] dithiolato/dithione (donor/acceptor) second-order nonlinear chromophores: Combined experimental and theoretical studies. Inorg. Chem..

[6] Espa D., Pilia L., Attar S., Serpe A., Deplano P. (2018). Molecular engineering of heteroleptic metal-dithiolene complexes with optimized second-order NLO response. Inorg. Chim. Acta.

[7] Zhao H., Garoni E., Roisnel T., Colombo A., Dragonetti C., Marinotto D., Righetto S., Roberto D., Jacquemin D., Boixel J. (2018). Photochromic DTE-substituted-1,3-di(2-pyridyl)benzene platinum(II) complexes: Photomodulation of luminescence and second-order nonlinear optical properties. Inorg. Chem..

[8] Fagnani F., Colombo A., Malandrino G., Dragonetti C., Pellegrino A.L. (2022). Luminescent 1,10-phenanthroline β-diketonate europium complexes with large second-order nonlinear optical properties. Molecules.

[9] Fagnani F., Colombo A., Dragonetti C., Roberto D., Guerchais V., Roisnel T., Marinotto D., Fantacci S. (2024). Multifunctional organometallic compounds: An interesting luminescent NLO-active alkynylplatinum (II) complex. Eur. J. Inorg. Chem..

[10] Colombo A., Dragonetti C., Fagnani F., Roberto D., Fantacci S. (2025). Dipolar copper(I) complexes: A novel appealing class of highly active second-order NLO-phores. Molecules.

[11] Chakraborty S., Wadas T.J., Hester H., Schmehl R., Eisenberg R. (2005). Platinum chromophore-based systems for photoinduced charge separation: A molecular design approach for artificial photosynthesis. Inorg. Chem..

[12] Yoshida M., Saito K., Matsukawa H., Yanagida S., Ebina M., Maegawa Y., Inagaki S., Kobayashi A., Kato M. (2018). Immobilization of luminescent Platinum(II) complexes on periodic mesoporous organosilica and their water reduction photocatalysis. J. Photochem. Photobiol. A.

[13] Domingo-Legarda P., Casado-Sánchez A., Marzo L., Alemán J., Cabrera S. (2020). Photocatalytic water-soluble cationic platinum(II) complexes bearing quinolinate and phosphine ligands. Inorg. Chem..

[14] Gómez de Segura D., Corral-Zorzano A., Alcolea E., Moreno M.T., Lalinde E. (2024). Phenylbenzothiazole-based platinum (II) and diplatinum (II) and (III) complexes with pyrazolate groups: Optical properties and photocatalysis. Inorg. Chem..

[15] Wenger O.S. (2013). Vapochromism in organometallic and coordination complexes: Chemical sensors for volatile organic compounds. Chem. Rev..

[16] Kobayashi A., Kato M. (2014). Vapochromic platinum(II) complexes: Crystal engineering toward intelligent sensing devices. Eur. J. Inorg. Chem..

[17] Rodrigue-Witchel A., Rochester D.L., Zhao S.-B., Lavelle K.B., Williams J.A.G., Wang S., Connick W.B., Reber C. (2016). Pressure-induced variations of MLCT and ligand-centered luminescence spectra in square-planar platinum(II) complexes. Polyhedron.

[18] Attar S., Espa D., Artizzu F., Mercuri M.L., Serpe A., Sessini E., Concas G., Congiu F., Marchiò L., Deplano P. (2016). Platinum–dithiolene monoanionic salt exhibiting multiproperties, including room-temperature proton-dependent solution luminescence. Inorg. Chem..

[19] Attar S., Espa D., Artizzu F., Pilia L., Serpe A., Pizzotti M., Di Carlo G., Marchiò L., Deplano P. (2017). Optically multiresponsive heteroleptic platinum dithiolene complex with proton-switchable properties. Inorg. Chem..

[20] Law A.S.-Y., Yeung M.C.-L., Yam V.W.-W. (2017). Arginine-rich peptide-induced supramolecular self-assembly of water-soluble anionic alkynylplatinum(II) complexes: A continuous and label-free luminescence assay for trypsin and inhibitor screening. ACS Appl. Mater. Interfaces.

[21] Attar S., Artizzu F., Marchiò L., Espa D., Pilia L., Casula M.F., Serpe A., Pizzotti M., Orbelli-Biroli A., Deplano P. (2018). Uncommon optical properties and silver-responsive turn-off/on luminescence in a Pt^II^ heteroleptic dithiolene complex. Chem. Eur. J..

[22] Zheng Q., Borsley S., Tu T., Cockroft S.L. (2020). Reversible stimuli-responsive chromism of a cyclometallated platinum(II) complex. Chem. Commun..

[23] Haque A., El Moll H., Alenezi K.M., Khan M.S., Wong W.Y. (2021). Functional materials based on cyclometalated platinum (II) β-diketonate complexes: A Review of structure–property relationships and applications. Materials.

[24] Poh W.C., Au-Yeung H.-L., Chan A.K.-W., Hong E.Y.-H., Cheng Y.-H., Leung M.-Y., Lai S.-L., Low K.-H., Yam V.W.-W. (2021). Cyclometalated platinum(II) complexes with donor-acceptor-containing bidentate ligands and their application studies as organic resistive memories. Chem. Asian J..

[25] Yam V.W.-W., Cheng Y.-H. (2022). Stimuli-responsive and switchable platinum(II) Complexes and their applications in memory storage. Bull. Chem. Soc. Japan.

[26] Ning Y.Y., Jin G.Q., Wang M.X., Gao S., Zhang J.L. (2022). Recent progress in metal-based molecular probes for optical bioimaging and biosensing. Curr. Opin. Chem. Biol..

[27] Chan C.W.-T., Law A.S.-Y., Yam V.W.-W. (2023). A luminescence assay in the red for the detection of hydrogen peroxide and glucose based on metal coordination polyelectrolyte-induced supramolecular self-assembly of alkynylplatinum(II) complexes. Chem. Eur. J..

[28] Chan C.W.-T., Chan K., Yam V.W.-W. (2023). Induced self-assembly and disassembly of alkynylplatinum(II) 2,6-bis(benzimidazol-2’-yl)pyridine complexes with charge reversal properties: “proof-of-principle” demonstration of ratiometric Förster resonance energy transfer sensing of pH. ACS Appl. Mater. Interfaces.

[29] Xu Y., Leung M.-Y., Yan L., Chen Z., Li P., Cheng Y.-H., Chan M.H.-Y., Yam V.W.-W. (2024). Synthesis, characterization, and resistive memory behaviors of highly strained cyclometalated platinum(II) nanohoops. J. Am. Chem. Soc..

[30] Cocchi M., Virgili D., Fattori V., Rochester D.L., Williams J.A.G. (2007). N^C^N-coordinated platinum(II) complexes as phosphorescent emitters in high-performance organic light-emitting devices. Adv. Funct. Mater..

[31] Che C.M., Kwok C.C., Lai S.W., Rausch A.F., Finkenzeller W.J., Zhu N.Y., Yersin H. (2010). Photophysical Properties and OLED Applications of Phosphorescent Platinum(II) Schiff Base Complexes. Chem. Eur. J..

[32] Kalinowski J., Fattori V., Cocchi M., Williams J.A.G. (2011). Light-emitting devices based on organometallic platinum complexes as emitters. Coord. Chem. Rev..

[33] Gildea L.F., Williams J.A.G., Buckley A. (2013). Iridium and platinum complexes for OLEDs. Organic Light-Emitting Diodes: Materials, Devices and Applications.

[34] Cheng G., Chow P.-K., Kui S.C.F., Kwok C.-C., Che C.-M. (2013). High-efficiency polymer light-emitting devices with robust phosphorescent platinum(II) emitters containing tetradentate dianionic O^N^C^N ligands. Adv. Mater..

[35] Hsu C.-W., Ly K.T., Lee W.-K., Wu C.-C., Wu L.C., Lee J.-J., Lin T.-C., Liu S.-H., Chou P.-T., Lee G.-H. (2016). Triboluminescence and metal phosphor for organic light-emitting diodes: Functional Pt(II) complexes with both 2-pyridylimidazol-2-ylidene and bipyrazolate chelates. ACS Appl. Mater. Interfaces.

[36] Cebrian C., Mauro M. (2018). Recent advances in phosphorescent platinum complexes for organic light-emitting diodes. Beilstein J. Org. Chem..

[37] Ly K.T., Chen-Cheng R.-W., Lin H.-W., Shiau Y.-J., Liu S.-H., Chou P.-T., Tsao C.-S., Huang Y.-C., Chi Y. (2017). Near-infrared organic light-emitting diodes with very high external quantum efficiency and radiance. Nat. Photonics.

[38] Chen W.-C., Sukpattanacharoen C., Chan W.-H., Huang C.-C., Hsu H.-F., Shen D., Hung W.-Y., Kungwan N., Escudero D., Lee C.-S. (2020). Modulation of Solid-State Aggregation of Square-Planar Pt(II) Based Emitters: Enabling Highly Efficient Deep-Red/Near Infrared Electroluminescence. Adv. Funct. Mater..

[39] Dragonetti C., Fagnani F., Marinotto D., di Biase A., Roberto D., Cocchi M., Fantacci S., Colombo A. (2020). First member of an appealing class of cyclometalated 1,3-di-(2-pyridyl)benzene platinum(II) complexes for solution-processable OLEDs. J. Mater Chem. C.

[40] Shafikov M.Z., Pander P., Zaytsev A.V., Daniels R., Martinscroft R., Dias F.B., Williams J.A.G., Kozhevnikov V.N. (2021). Extended ligand conjugation and dinuclearity as a route to efficient platinum-based near-infrared (NIR) triplet emitters and solution-processed NIR-OLEDs. J. Mater. Chem. C.

[41] Colombo A., De Soricellis G., Fagnani F., Dragonetti C., Cocchi M., Carboni B., Guerchais V., Marinotto D. (2022). Introduction of a triphenylamine substituent on pyridyl rings as a springboard for a new appealing brightly luminescent 1,3-di-(2-pyridyl)benzene platinum(ii) complex family. Dalton Trans..

[42] Fagnani F., Colombo A., Dragonetti C., Roberto D., Marinotto D. (2022). The intriguing effect of thiolates as co-ligands in platinum(II) complexes bearing a cyclometalated 1,3-di(2-pyridyl)benzene. Inorg. Chim. Acta.

[43] Roberto D., Colombo A., Dragonetti C., Fagnani F., Cocchi M., Marinotto D. (2022). Novel class of cyclometalated platinum(II) complexes for solution-processable OLEDs. Molecules.

[44] Colombo A., De Soricellis G., Dragonetti C., Fagnani F., Roberto D., Carboni B., Guerchais V., Roisnel T., Cocchi M., Fantacci S. (2024). Introduction of a mesityl substituent on pyridyl rings as a facile strategy for improving the performance of luminescent 1,3-bis-(2-pyridyl)benzene platinum( ii ) complexes: A springboard for blue OLEDs. J. Mater. Chem. C.

[45] Zhou F., Pan Y., Hung W.-Y., Chen C.-F., Chen K.-M., Li J.-L., Yiu S.-M., Liu Y.-M., Chou P.-T., Chi Y. (2024). Tetradentate Pt(II) complexes based on xylenylamino linked dual pyrazolate chelates for organic light emitting diodes. Chem. Eur. J..

[46] Hung C.-M., Wang S.-F., Chao W.-C., Li J.-L., Chen B.-H., Lu C.-H., Tu K.-Y., Yang S.-D., Hung W.-Y., Chi Y. (2024). High-performance near-infrared OLEDs maximized at 925 nm and 1022 nm through interfacial energy transfer. Nat Commun..

[47] Zhao Q., Huang C., Li F. (2011). Phosphorescent heavy-metal complexes for bioimaging. Chem. Soc. Rev..

[48] Baggaley E., Weinstein J.A., Williams J.A.G. (2012). Lighting the way to see inside the live cell with luminescent transition metal complexes. Coord. Chem. Rev..

[49] Baggaley E., Botchway S.W., Haycock J.W., Morris H., Sazanovich I.V., Williams J.A.G., Weinstein J.A. (2014). Long-lived metal complexes open up microsecond lifetime imaging microscopy under multiphoton excitation: From FLIM to PLIM and beyond. Chem. Sci..

[50] Mauro M., Aliprandi A., Septiadi D., Kehr N.S., De Cola L. (2014). When self-assembly meets biology: Luminescent platinum complexes for imaging applications. Chem. Soc. Rev..

[51] Colombo A., Fiorini F., Septiadi D., Dragonetti C., Nisic F., Valore A., Roberto D., Mauro M., De Cola L. (2015). Neutral N^C^N terdentate luminescent Pt(II) complexes: Their synthesis, photophysical properties, and bio-imaging applications. Dalton Trans..

[52] Mitra K., Lyons C.E., Hartman M.C.T. (2018). A platinum(II) complex of heptamethine cyanine for photoenhanced cytotoxicity and cellular imaging in near-IR light. Angew. Chem. Int. Ed..

[53] Yam V.W.-W., Law A.S.-Y. (2020). Luminescent d8 metal complexes of platinum(II) and gold(III): From photophysics to photofunctional materials and probes. Coord. Chem. Rev..

[54] Law A.S.-Y., Lee L.C.-C., Lo K.K.-W., Yam V.W.-W. (2021). Aggregation and supramolecular self-assembly of low-energy red luminescent alkynylplatinum(II) complexes for RNA detection, nucleolus imaging, and RNA synthesis inhibitor screening. J. Am. Chem. Soc..

[55] Li B., Wang Y., Chan M.H.-Y., Pan M., Li Y., Yam V.W.-W. (2022). Supramolecular Assembly of Organoplatinum(II) Complexes for Subcellular Distribution and Cell Viability Monitoring with Differentiated Imaging. Angew. Chem..

[56] Ai Y., Zhang Z., Fei Y., Ye R., Law A.S.-Y., Mao Z.-W., Liu J., Li Y., Yam V.W.-W. (2023). Rational design of platinum(II) complexes with orthogonally oriented triazolyl ligand with emission enhancement characteristics for cancer chemotherapy in vivo. Sci. China.

[57] Berrones Reyes J., Sherin P.S., Sarkar A., Kuimova M.K., Vilar R. (2023). Platinum(II)-based optical probes for imaging quadruplex DNA structures via phosphorescence lifetime imaging microscopy. Angew. Chem. Int. Ed..

[58] Wu J., Xu B., Xu Y., Yue L., Chen J., Xie G., Zhao J. (2023). Reblooming of the cis-bis(2-phenylpyridine) platinum(II) complex: Synthesis updating, aggregation-induced emission, electroluminescence, and cell imaging. Inorg. Chem..

[59] Clancy E., Ramadurai S., Needham S.R., Baker K., Eastwood T.A., Weinstein J.A., Mulvihill D.P., Botchway S.W. (2023). Fluorescence and phosphorescence lifetime imaging reveals a significant cell nuclear viscosity and refractive index changes upon DNA damage. Sci. Rep..

[60] Doherty R.E., Sazanovich I.V., McKenzie L.K., Stasheuski A.S., Coyle R., Baggaley E., Bottomley S., Weinstein J.A., Bryant H.E. (2016). Photodynamic killing of cancer cells by a platinum(II) complex with cyclometallating ligand. Sci. Rep..

[61] Chatzisideri T., Thysiadis S., Katsamakas S., Dalezis P., Sigala I., Lazarides T., Nikolakaki E., Trafalis D., Gederaas O.A., Lindgren M. (2017). Synthesis and biological evaluation of a platinum(II)-c(RGDyK) conjugate for integrin-targeted photodynamic therapy. Eur. J. Med. Chem..

[62] Shi H., Clarkson G.J., Sadler P.J. (2019). Dual action photosensitive platinum(II) anticancer prodrugs with photoreleasable.azide ligands. Inorg. Chim. Acta.

[63] McKenzie L.K., Bryant H.E., Weinstein J.A. (2019). Transition metal complexes as photosensitisers in one- and two-photon photodynamic therapy. Coord. Chem. Rev..

[64] Scoditti S., Dabbish E., Russo N., Mazzone G., Sicilia E. (2021). Anticancer activity, DNA binding, and photodynamic properties of a N^C^N-coordinated Pt(II) complex. Inorg. Chem..

[65] De Soricellis G., Fagnani F., Colombo A., Dragonetti C., Roberto D. (2022). Exploring the potential of N^C^N cyclometalated Pt(II) complexes bearing 1,3-di(2-pyridyl)benzene derivatives for imaging and photodynamic therapy. Inorg. Chim. Acta.

[66] Fagnani F., De Soricellis G., Colombo A., Dragonetti C., Roberto D., di Biase A., Fantacci S., Marinotto D. (2024). Photophysical investigation of highly phosphorescent N^C^N platinum(II) azido complexes and their triazole derivatives. Dye. Pigment..

[67] Upadhyay A., Nepalia A., Bera A., Saini D.K., Chakravarty A.R. (2023). A Platinum(II) boron-dipyrromethene complex for cellular imaging and mitochondria-targeted photodynamic therapy in red light. Chem. Asian J..

[68] Yersin H., Rausch A.F., Czerwieniec R., Hofbeck T., Fischer T. (2011). The triplet state of organo-transition metal compounds. Triplet harvesting and singlet harvesting for efficient OLEDs. Coord. Chem. Rev..

[69] Chou P.T., Chi Y., Chung M.W., Lin C.C. (2011). Harvesting luminescence via harnessing the photophysical properties of transition metal complexes. Coord. Chem. Rev..

[70] Cocchi M., Kalinowski J., Fattori V., Williams J.A.G., Murphy L. (2009). Color-variable highly efficient organic electrophosphorescent diodes manipulating molecular exciton and excimer emissions. Appl. Phys. Lett..

[71] Williams J.A.G. (2009). The coordination chemistry of dipyridylbenzene: N-deficient terpyridine or panacea for brightly luminescent metal complexes?. Chem. Soc. Rev..

[72] Williams J.A.G., Beeby A., Davies E.S., Weinstein J.A., Wilson C. (2003). An alternative route to highly luminescent platinum(II) complexes:  cyclometalation with N∧C∧N-coordinating dipyridylbenzene ligands. Inorg. Chem..

[73] Williams J.A.G. (2007). Photochemistry and Photophysics of Coordination Compounds: Platinum. Top. Curr. Chem..

[74] Mdleleni M.M., Bridgewater J.S., Watts R.J., Ford P.C. (1995). Synthesis, structure, and spectroscopic properties of ortho-metalated platinum(II) complexes. Inorg. Chem..

[75] Sotoyama W., Satoh T., Sato H., Matsuura A., Sawatari N. (2005). Excited states of phosphorescent platinum(II) complexes containing N^C^N-coordinating tridentate ligands:  Spectroscopic investigations and time-dependent density functional theory calculations. J. Phys. Chem. A.

[76] Cocchi M., Kalinowski J., Murphy L., Williams J.A.G., Fattori V. (2010). Mixing of molecular exciton and excimer phosphorescence to tune color and efficiency of organic LEDs. Org. Electron..

[77] Shen Y., Kong X., Yang F., Bian H.-D., Cheng G., Cook T.R., Zhang Y. (2022). Deep Blue Phosphorescence from Platinum Complexes Featuring Cyclometalated N-Pyridyl Carbazole Ligands with Monocarborane Clusters (CB11H12−). Inorg. Chem..

[78] Maganti T., Venkatesan K. (2022). The Search for Efficient True Blue and Deep Blue Emitters: An Overview of Platinum Carbene Acetylide Complexes. ChemPlusChem.

[79] Nguyen Y.H., Dang V.Q., Soares J.V., Wu J.I., Teets T.S. (2023). Efficient blue-phosphorescent trans-bis(acyclic diaminocarbene) platinum(ii) acetylide complexes. Chem. Sci..

[80] Rausch A.F., Murphy L., Williams J.A.G., Yersin H. (2012). Improving the Performance of Pt(II) Complexes for Blue Light Emission by Enhancing the Molecular Rigidity. Inorg. Chem..

[81] Suzuki K., Kobayashi A., Kaneko S., Takehira K., Yoshihara T., Ishida H., Shiina Y., Oishic S., Tobita S. (2009). Reevaluation of absolute luminescence quantum yields of standard solutions using a spectrometer with an integrating sphere and a back-thinned CCD detector. Phys. Chem. Chem. Phys..

