# Supplementary material for: Effect of the Substitution of the Mesityl Group with Other Bulky Substituents on the Luminescence Performance of [Pt(1,3-bis(4-Mesityl-pyridin-2-yl)-4,6-difluoro-benzene)Cl]
Source: Molecules. 2025 Mar 27;30(7):1498. doi: 10.3390/molecules30071498 (PMC11990200; doi:10.3390/molecules30071498)
Supplement: Supplementary file 1 [file molecules-30-01498-s001.zip › molecules-3531332-supplementary.pdf]

Effect of the substitution of the mesityl group with other bulky substituents on the luminescence performance of [Pt(1,3-bis(4-mesityl-pyridin-2-yl)-4,6-difluoro-benzene)Cl]

Giulia De Soricellis,[a,b] Véronique Guerchais,[c] Alessia Colombo,[a] Claudia Dragonetti,[a] Francesco Fagnani,[a,*] Dominique Roberto,[a] and Daniele Marinotto[d]

[a] Dipartimento di Chimica, Università degli Studi di Milano and UdR-INSTM di Milano, Via C. Golgi 19, I-20133 Milan, Italy.

[b] Dipartimento di Chimica, Università di Pavia, Via Taramelli 12, I-27100 Pavia, Italy.

[c] Université de Rennes 1, CNRS, ISCR-UMR 6226, F-35000 Rennes, France.

[d] Istituto di Scienze e Tecnologie Chimiche (SCITEC) "Giulio Natta", Consiglio Nazionale delle Ricerche (CNR), via C. Golgi 19, I-20133 Milan, Italy.

^{1}H, ^{13}C, and ^{19}F NMR spectra - [PtL^{2}Cl]

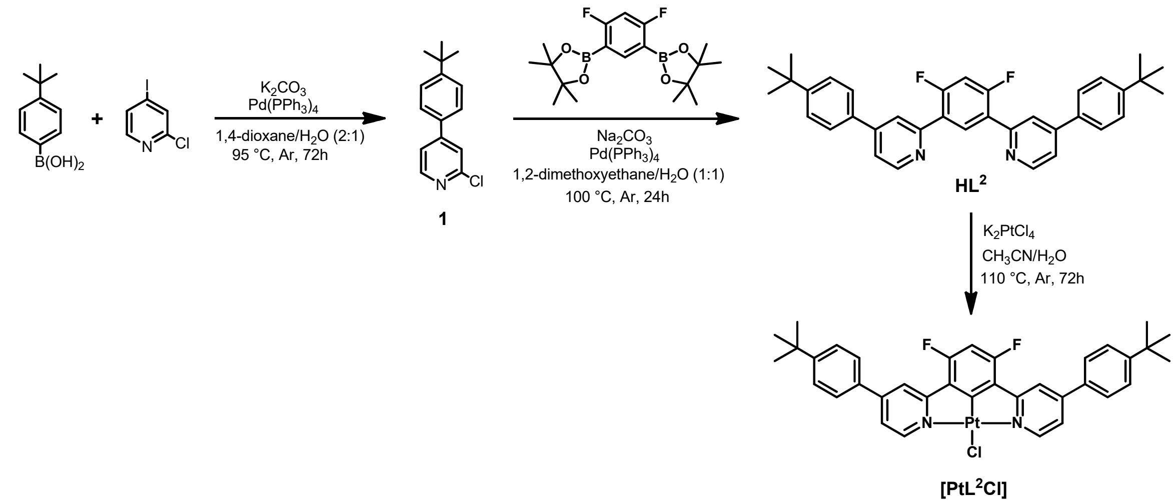

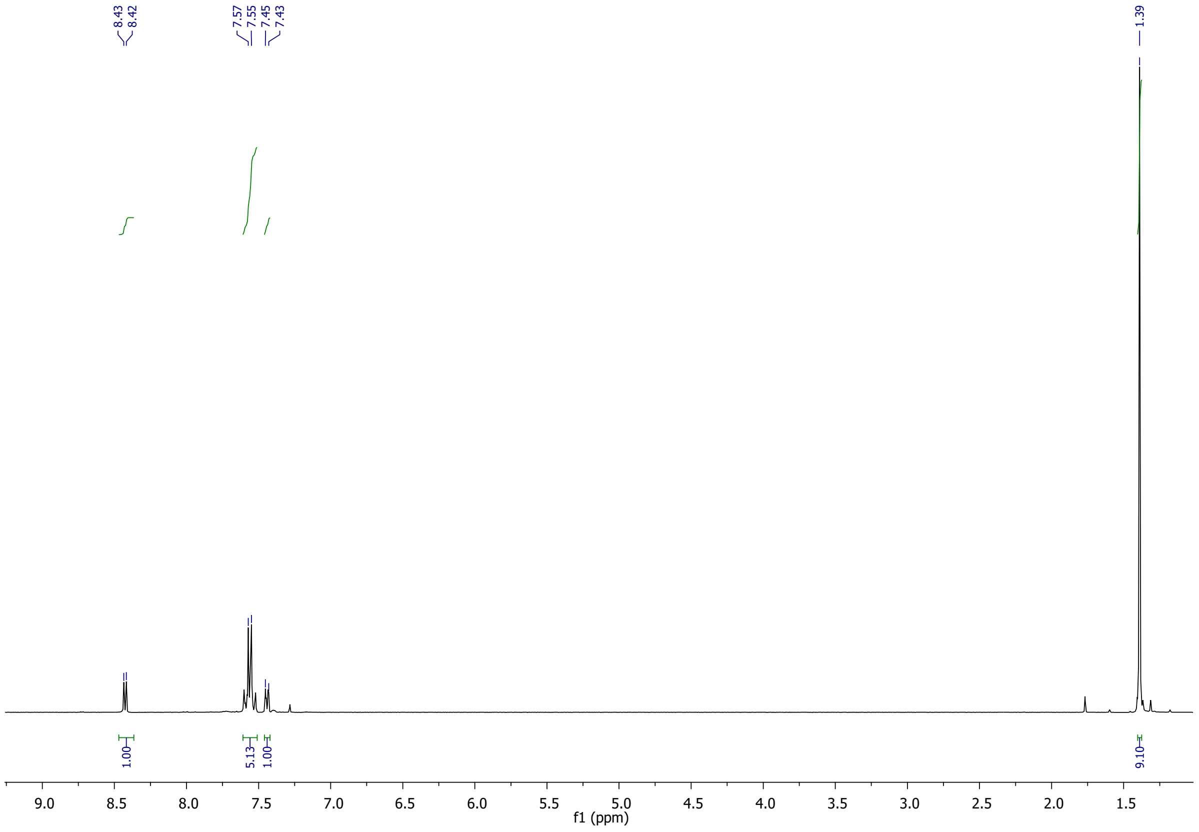

^{1}H NMR spectrum ($CDCl_3$, 300 MHz) of compound **1**.

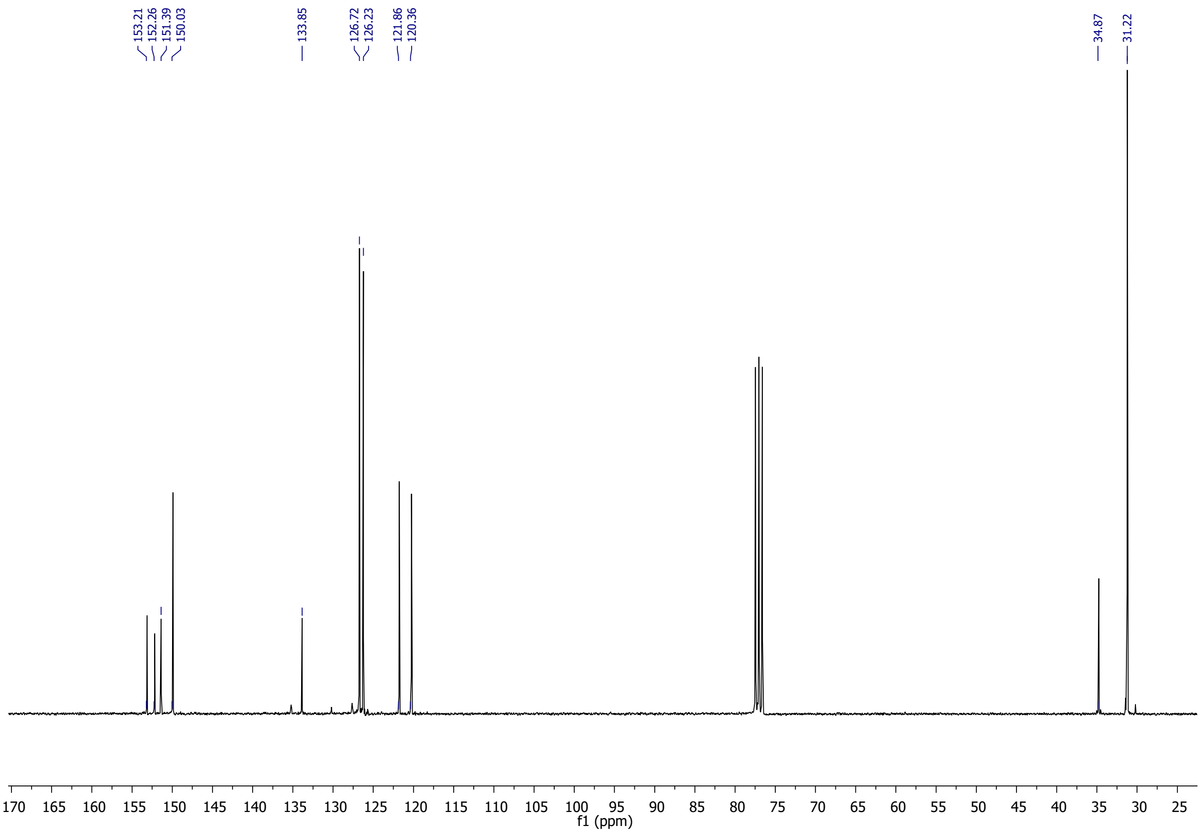

$^{13}C\{^{1}H\}$ NMR spectrum ($CDCl_3$, 75.48 MHz) of compound **1**.

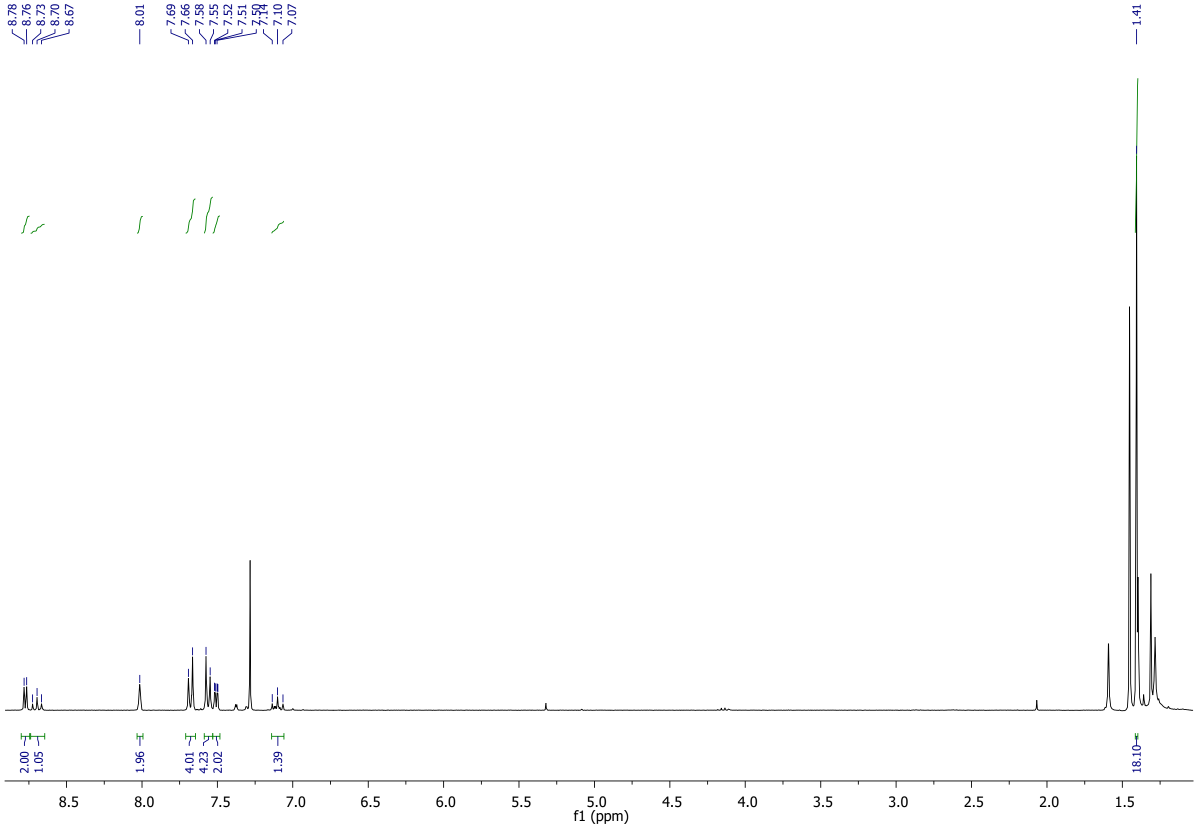

^{1}H NMR spectrum ($CDCl_3$, 300 MHz) of compound $\mathbf{HL^2}$.

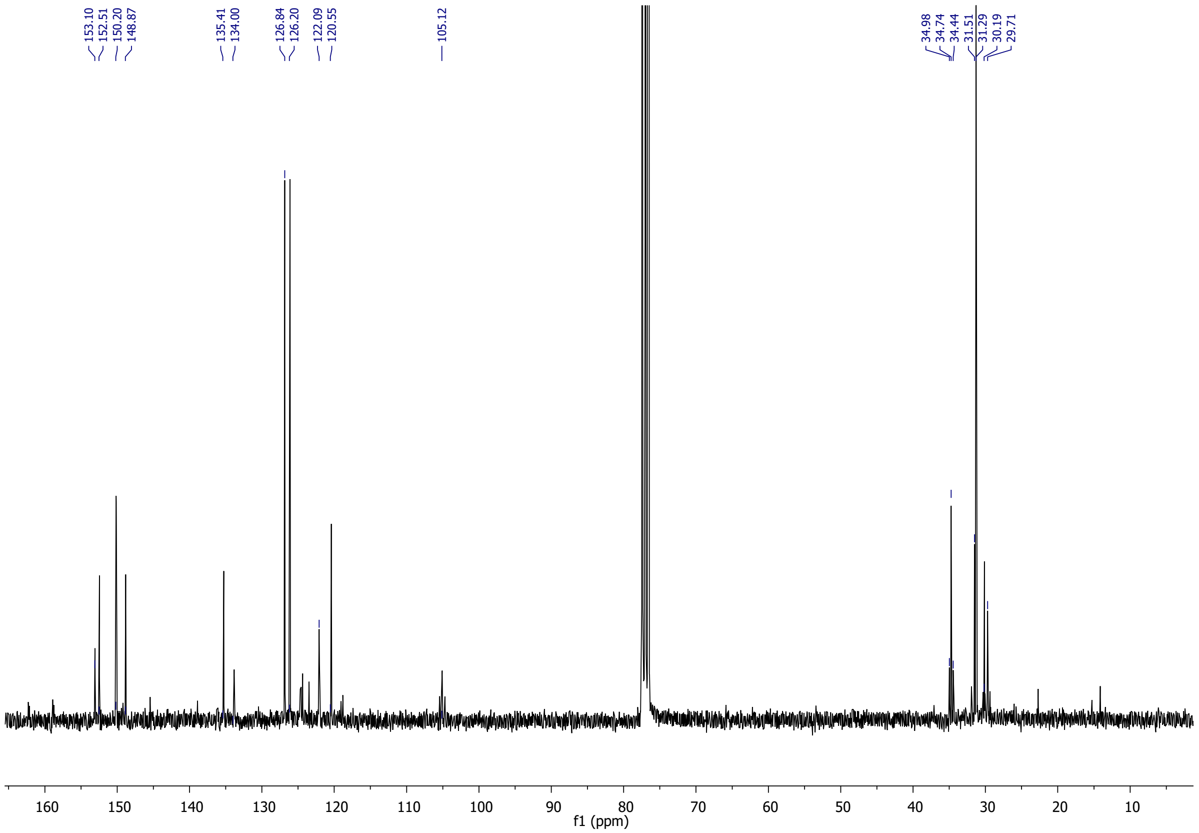

$^{13}C\{^1H\}$ NMR spectrum ($CDCl_3$, 75.48 MHz) of compound **HL^2**.

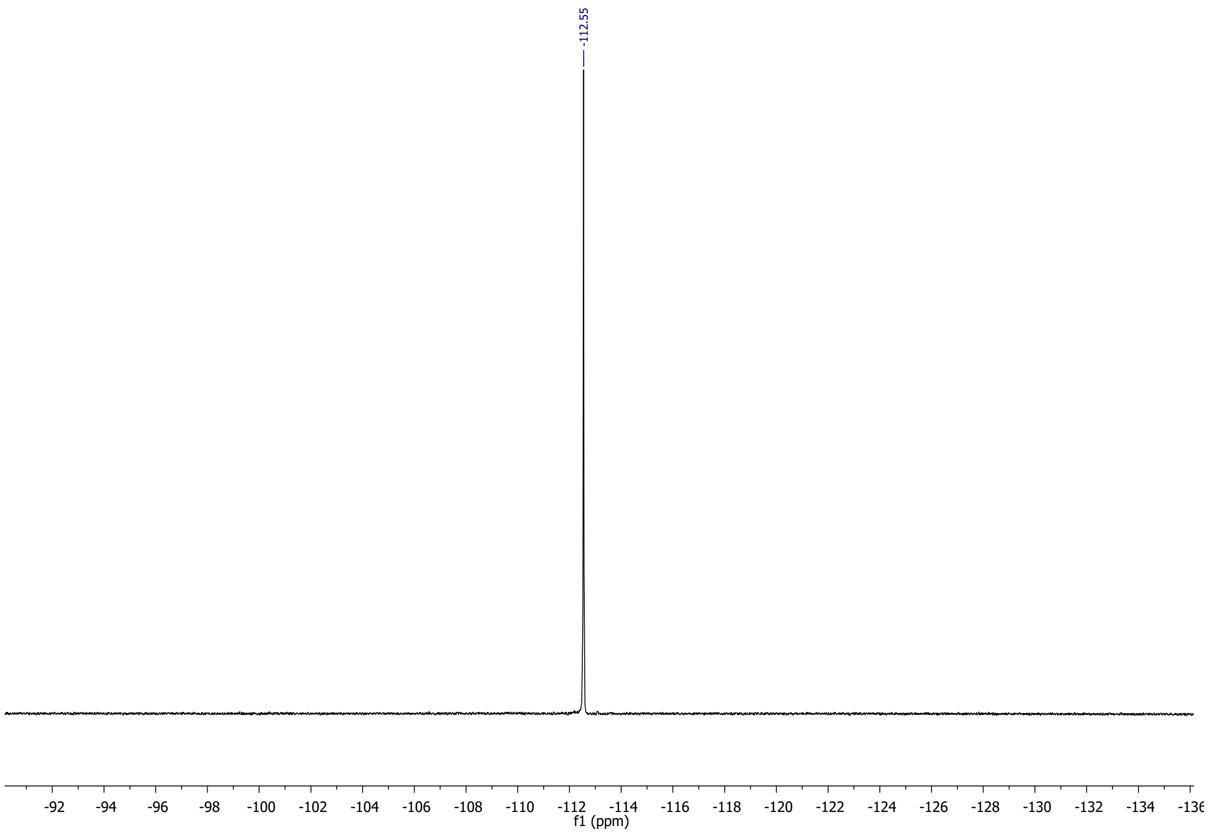

$^{19}F\{^{1}H\}$ NMR spectrum ($CDCl_3$, 282.36 MHz) of compound **HL^2**.

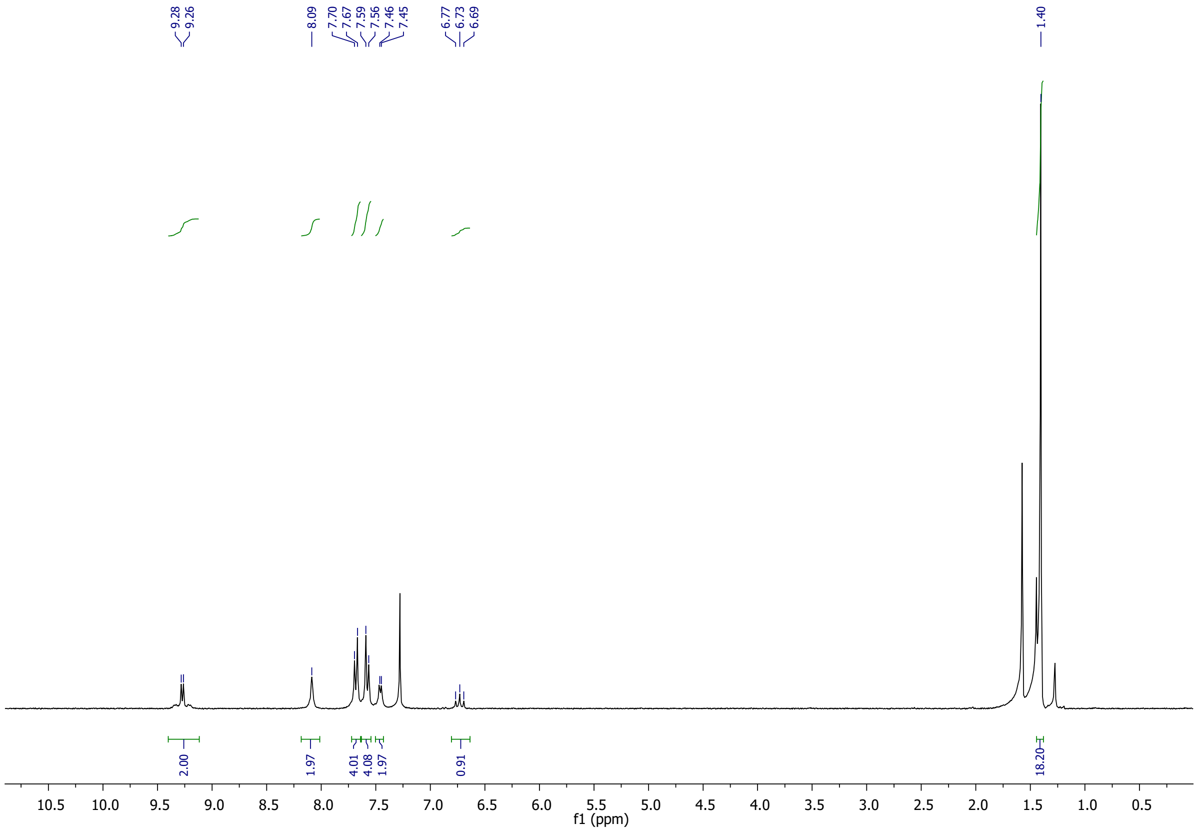

^{1}H NMR spectrum ($CDCl_3$, 300 MHz) of **[PtL^{2}Cl]**.

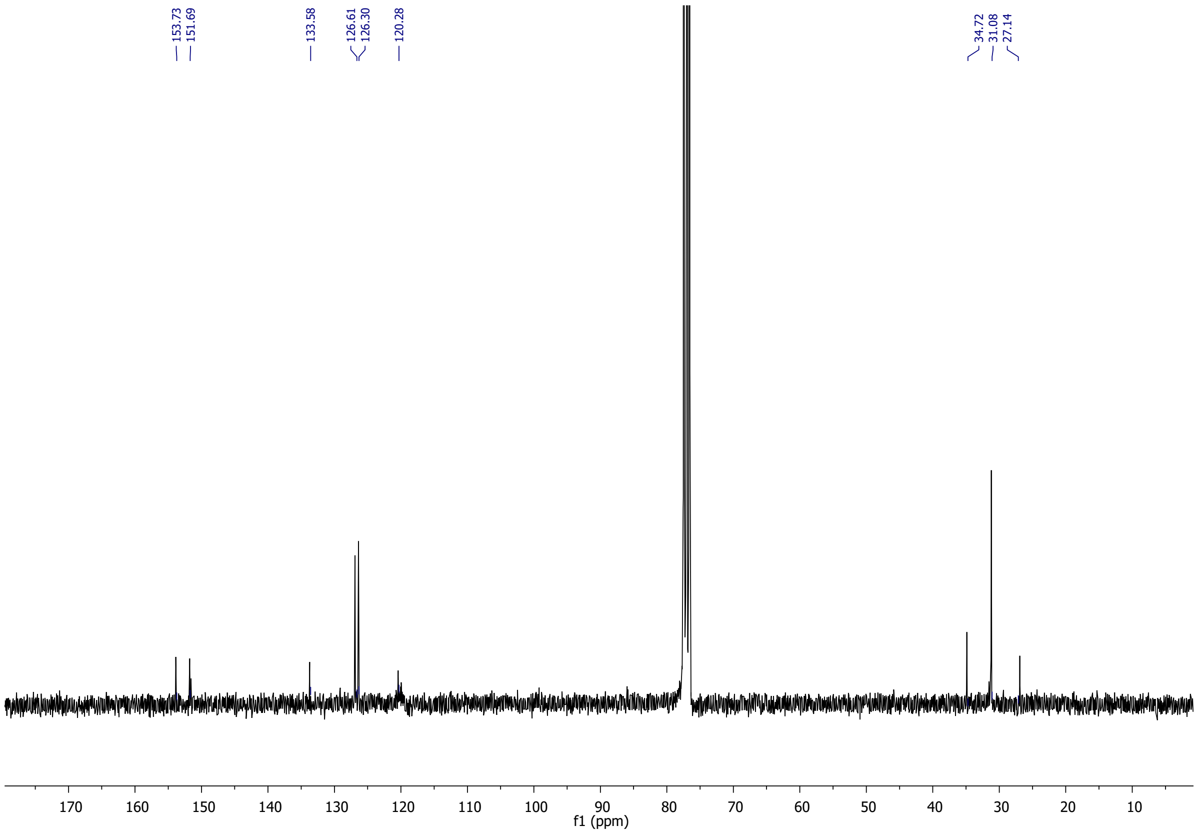

$^{13}C\{^{1}H\}$ NMR spectrum ($CDCl_3$, 75.48 MHz) of compound **[PtL²Cl]**.

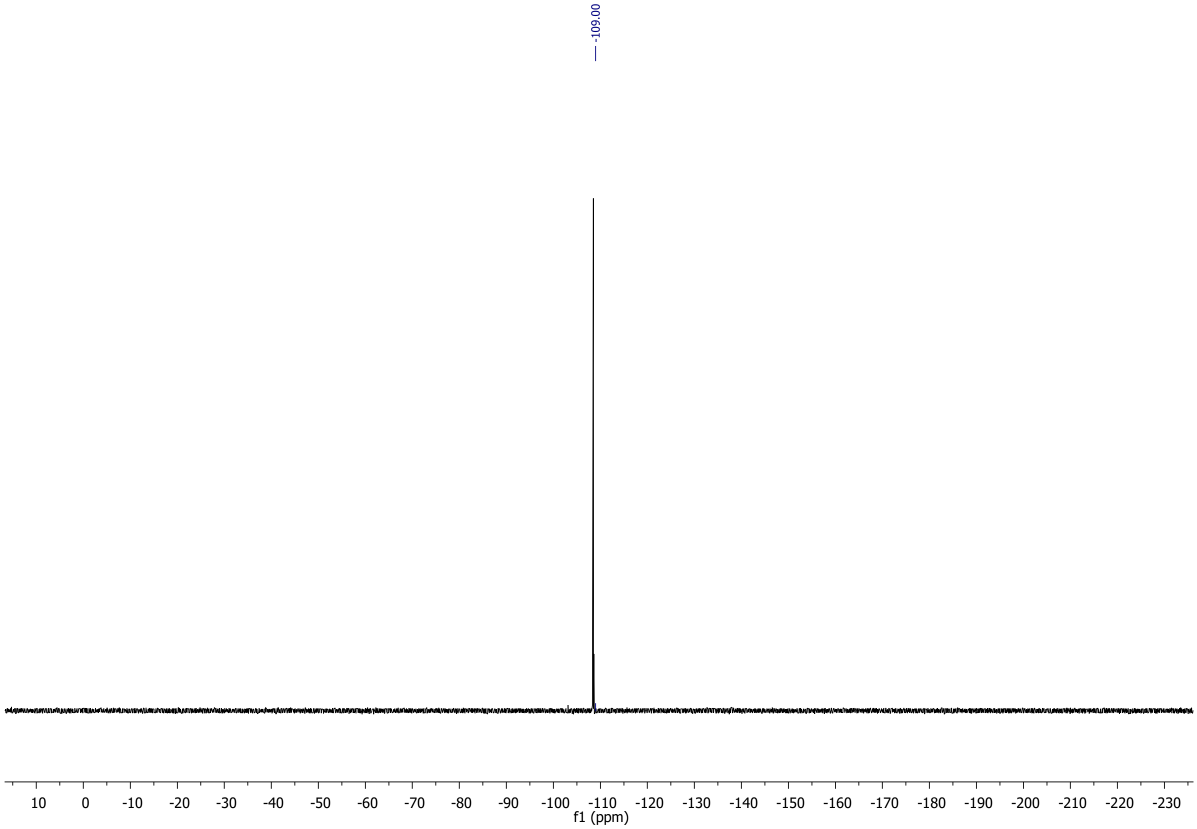

$^{19}F\{^{1}H\}$ NMR spectrum ($CDCl_3$, 282.36 MHz) of compound **[PtL^{2}Cl].**

^{1}H, ^{13}C, and ^{19}F NMR spectra - [PtL^{3}Cl]

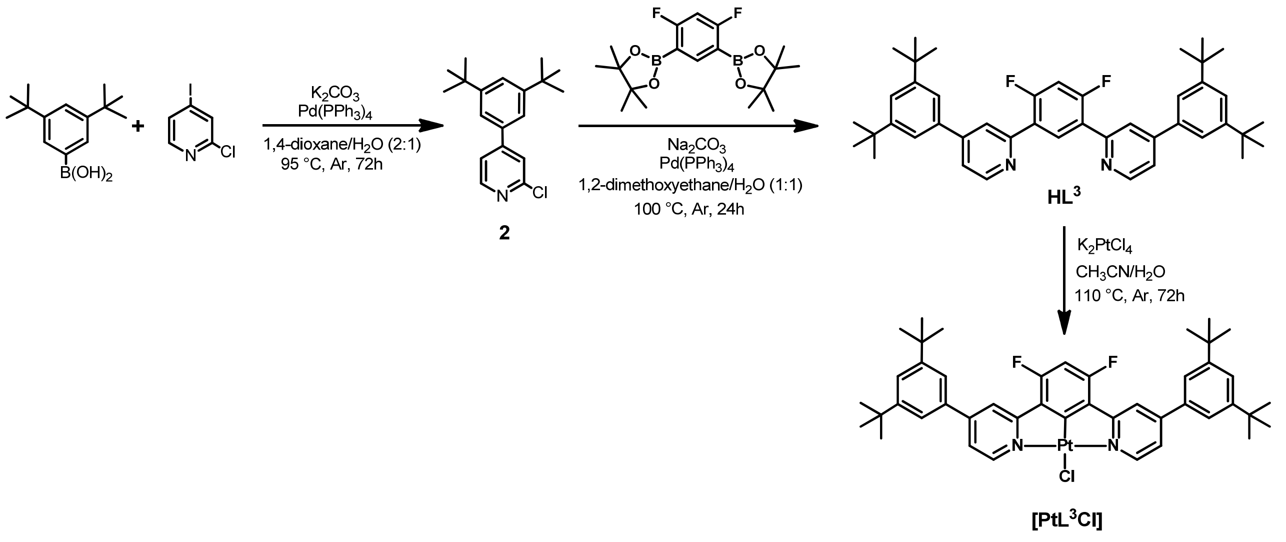

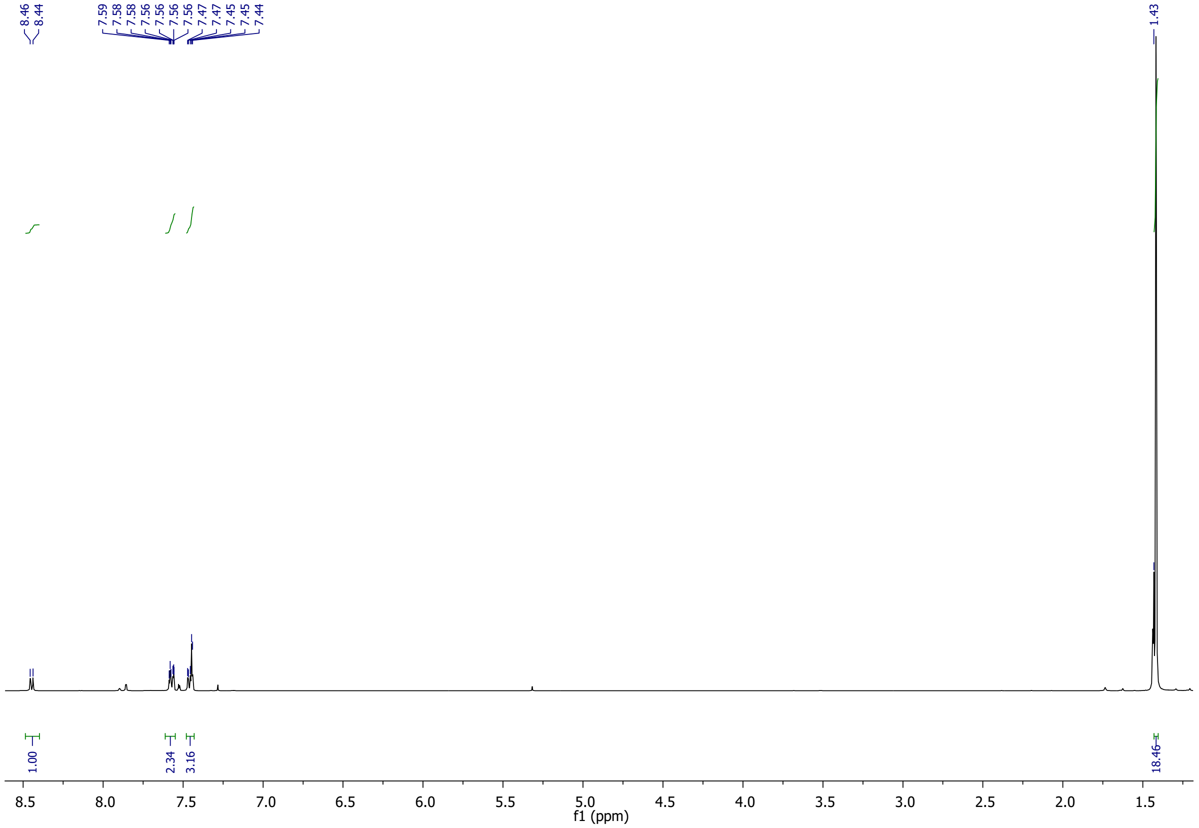

^{1}H NMR spectrum ($CDCl_3$, 300 MHz) of compound **2**.

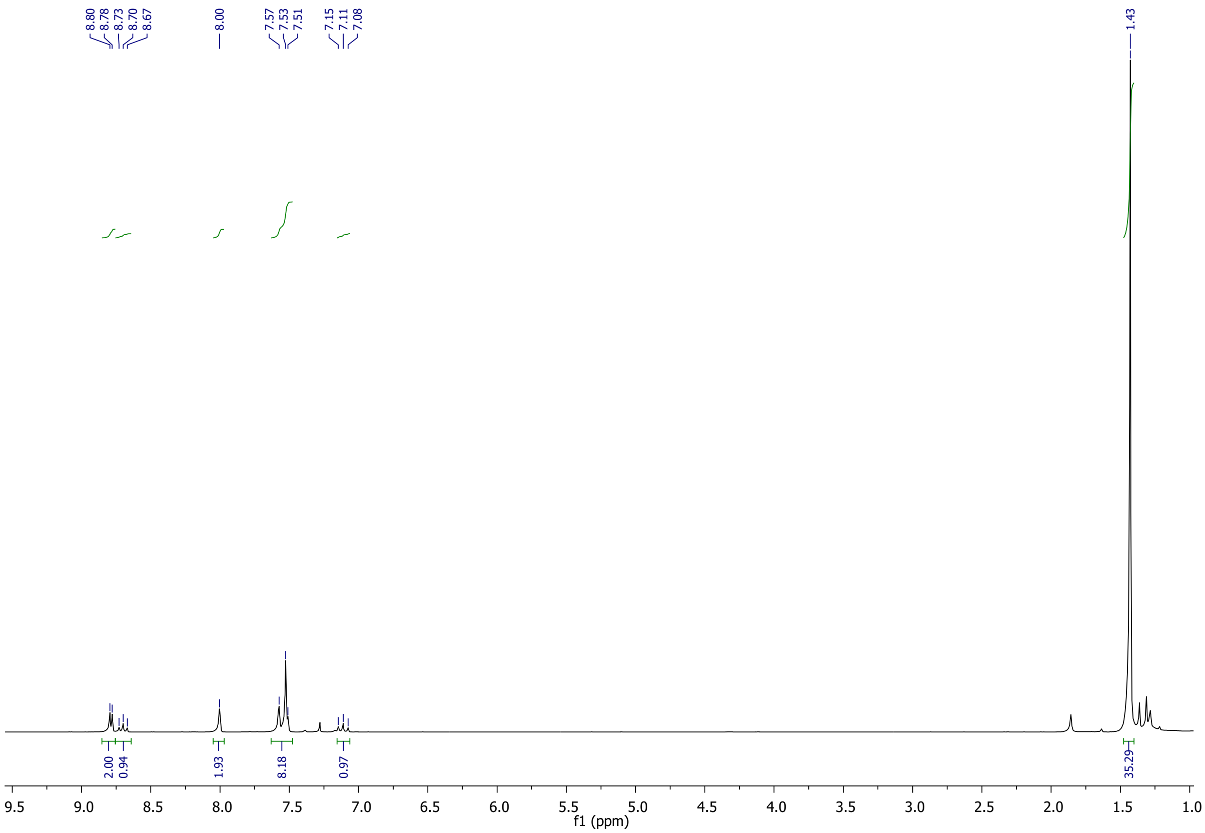

^{1}H NMR spectrum ($CDCl_3$, 300 MHz) of compound **HL[3]**.

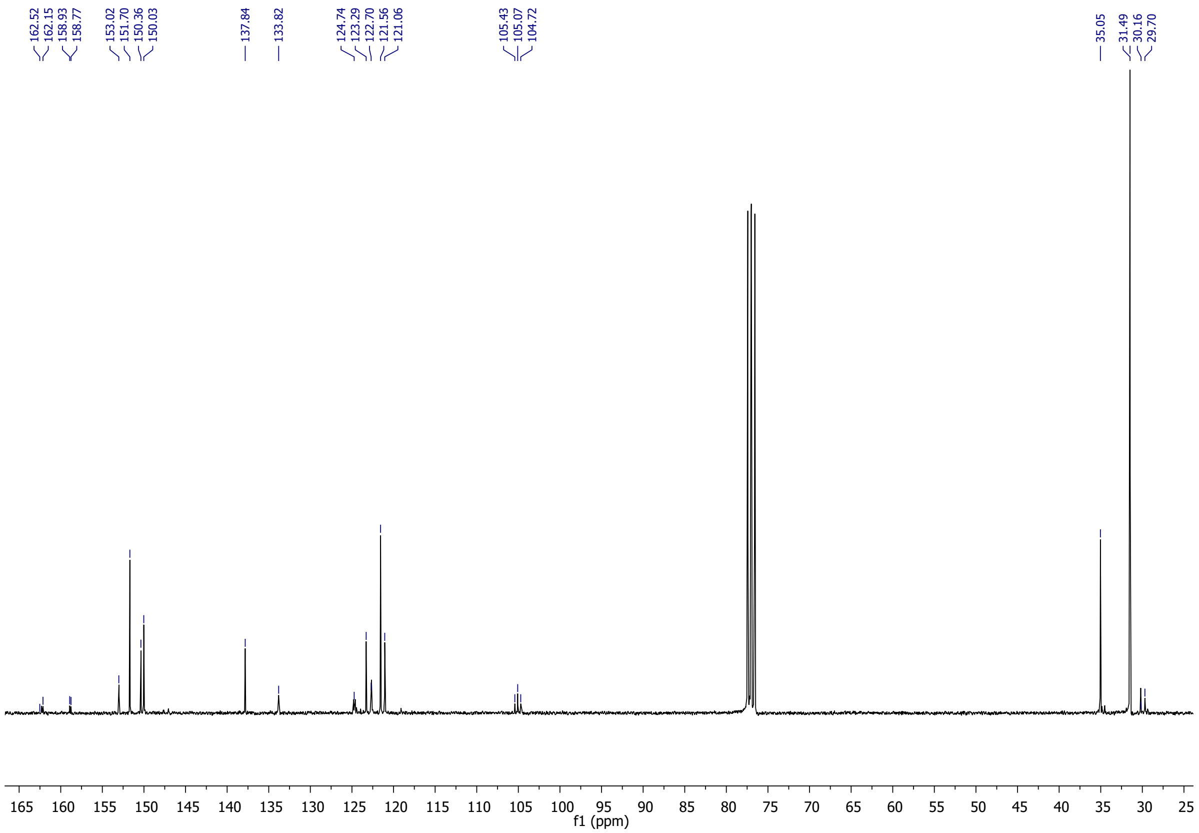

$^{13}C\{^{1}H\}$ NMR spectrum ($CDCl_3$, 75.48 MHz) of compound **HL^3**.

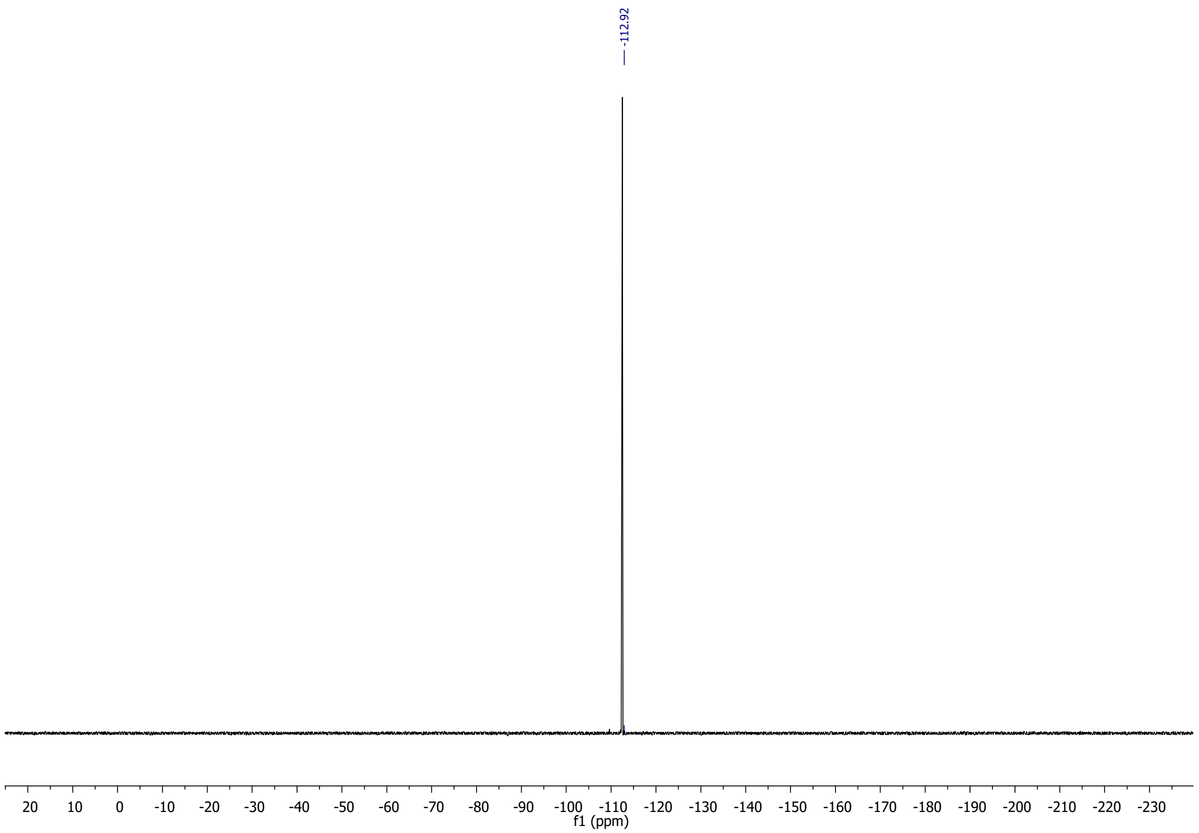

$^{19}F\{^{1}H\}$ NMR spectrum ($CDCl_3$, 282.36 MHz) of compound **HL^{3}**.

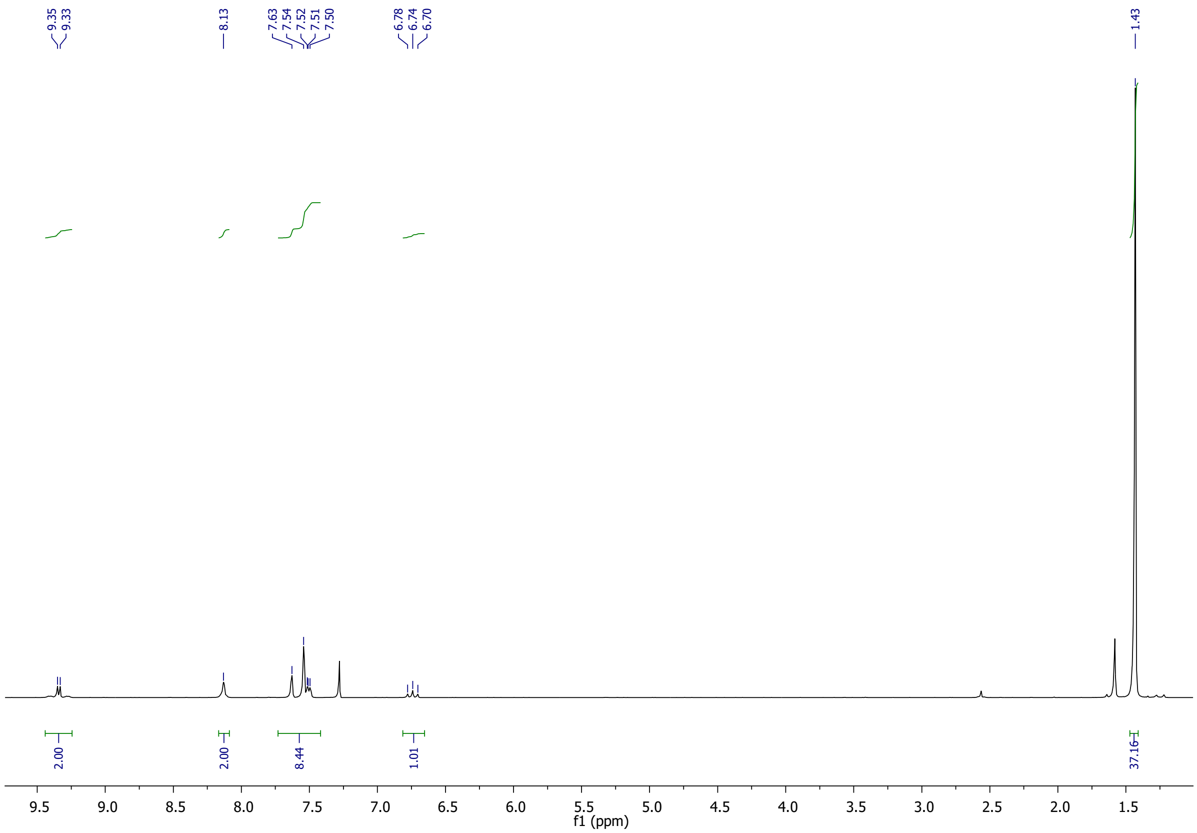

^{1}H NMR spectrum ($CDCl_3$, 300 MHz) of **[PtL[3]Cl]**.

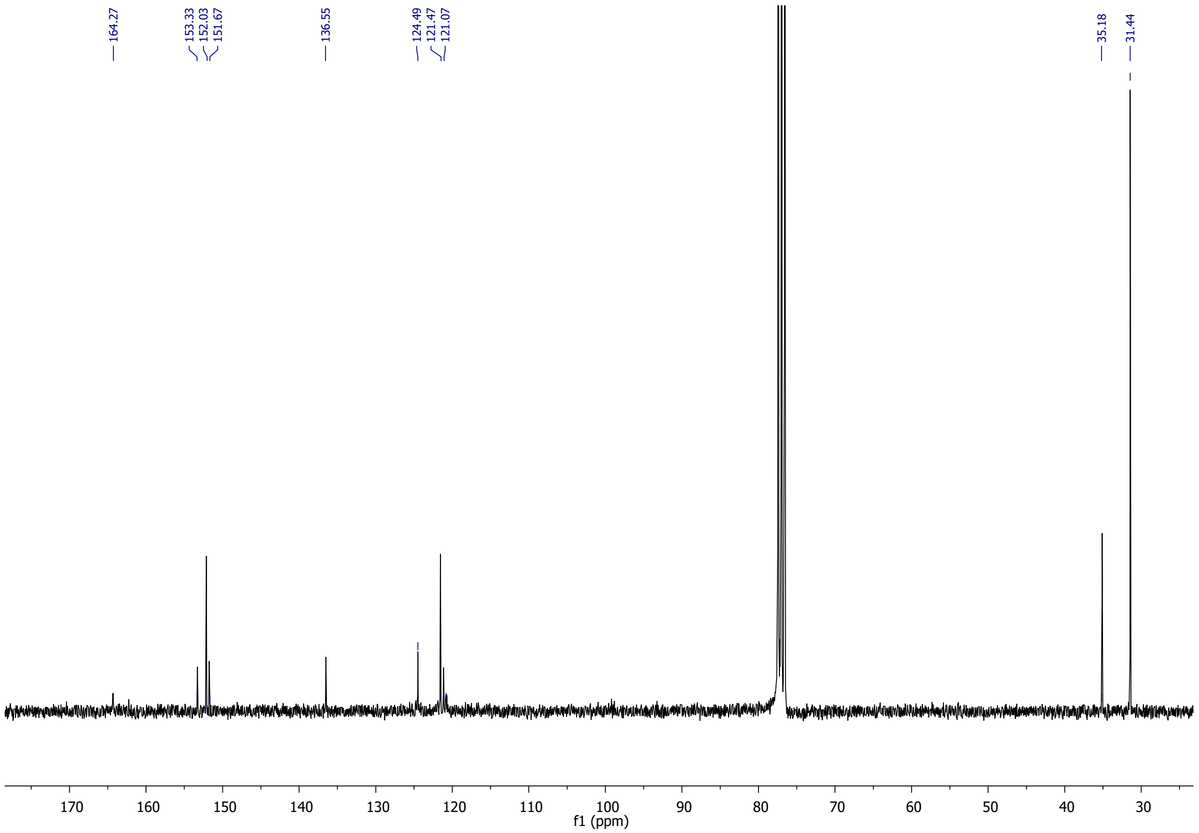

$^{13}C\{^{1}H\}$ NMR spectrum ($CDCl_3$, 75.48 MHz) of compound **[PtL^{3}Cl]**.

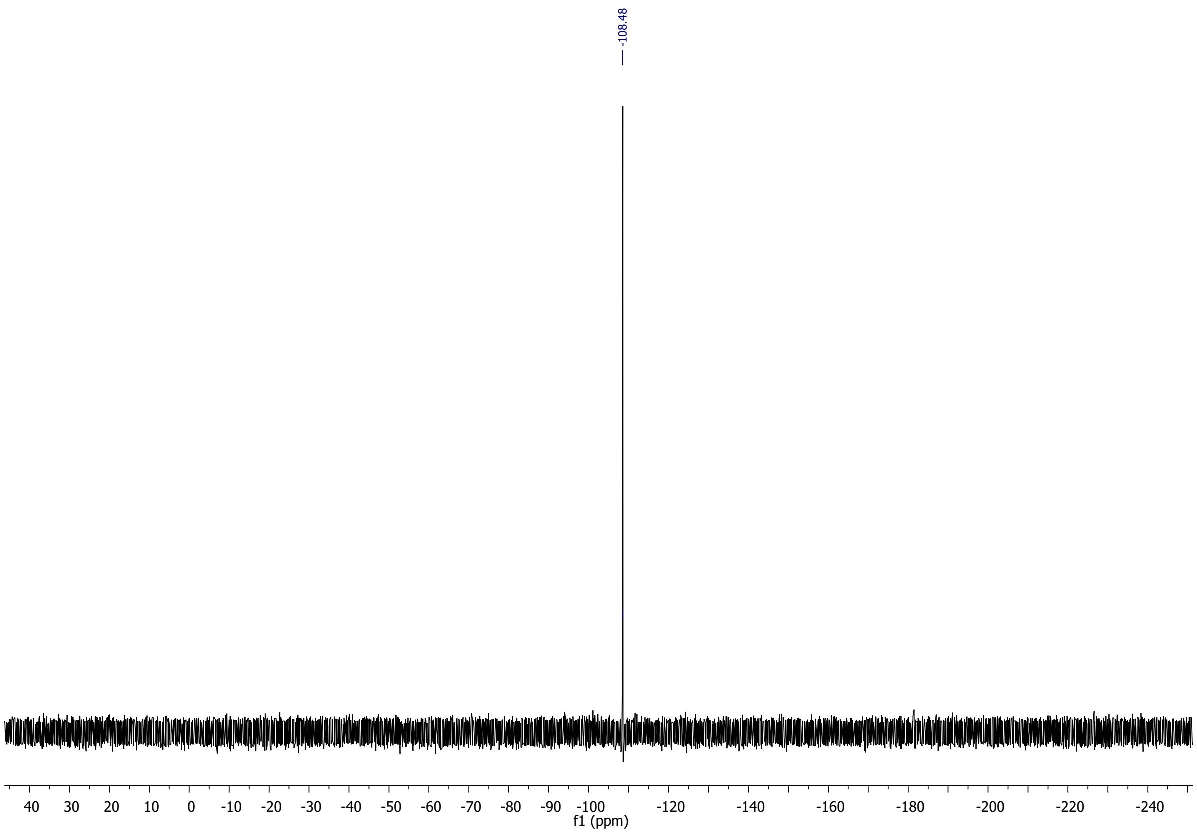

$^{19}F\{^{1}H\}$ NMR spectrum ($CDCl_3$, 282.36 MHz) of compound **[PtL^{3}Cl]**.

General comments about photophysical characterizations

Solutions were sonicated for 20 minutes before photophysical characterizations.
UV-Visible spectra were collected with a Shimadzu UV3600 spectrophotometer.
Luminescence measurements were carried out in CH_2Cl_2 solution after the Freeze-Pump-Thaw (FPT) procedure, necessary to remove dissolved oxygen.
Absolute photoluminescence quantum yield, Φ, was measured using a C11347 Quantaurus Hamamatsu Photonics K.K spectrometer. A description of the experimental setup and measurement method can be found in the article of K. Suzuki *et al.*[1]
Φ was calculated through Equation:

$$\Phi = \frac{PN\ (Em)}{PN\ (Abs)} = \frac{\int \frac{\lambda}{hc}\left[I_{em}^{sample}(\lambda) - I_{em}^{reference}(\lambda)\right]d\lambda}{\int \frac{\lambda}{hc}\left[I_{exc}^{sample}(\lambda) - I_{exc}^{reference}(\lambda)\right]d\lambda}$$

where PN(Em) is the number of emitted photons, PN(Abs) the number of absorbed photons, λ the wavelength, h the Planck's constant, c the speed of light, $I_{em}^{sample}$and $I_{em}^{reference}$ the photoluminescence intensities of the sample solution and reference in CH_2Cl_2, I_{exc}^{sample} and $I_{exc}^{reference}$ the excitation light intensities of the sample solution and reference in CH_2Cl_2. PN(Em) is calculated in the wavelength interval [λ_i, λ_f], where λ_i is taken 10 nm below the excitation wavelength, while λ_f is the upper end wavelength in the emission spectrum.
Steady state and time-resolved fluorescence data were obtained using a FLS980 spectrofluorimeter (Edinburg Instrument Ltd). Emission spectra were corrected for background intensity and quantum efficiency of the photomultiplier tube. Excitation spectra were corrected for the intensity fluctuation of a 450 W Xenon arc lamp. Quartz cuvettes with 1 cm optical path length were used for diluted solution, meanwhile quartz cuvettes of 1 mm optical path length were used for concerted solution. Time-resolved fluorescence measurements were performed through the time-correlated single photon counting technique with an Edinburgh Picosecond Pulsed Diode Laser (emitted wavelength 374 nm). Moreover, time-resolved fluorescence curves were fitted using an exponential function:

$$I(\lambda, t) = \alpha(\lambda) \exp\left(\frac{-t}{\tau}\right)$$

where α (λ) is the amplitude at wavelength λ and τ is the lifetime. The quality of the fit was evaluated through the reduced χ^2 values.

Photophysical characterization of [PtL^{2}Cl]

UV-Vis absorption

Table S1. Molar extinction coefficients for **[PtL^{2}Cl]** at different wavelengths.

λ	280 nm	312 nm	341 nm	383 nm	470 nm
ε / M^{-1} cm^{-1}	49781	38482	17328	23364	310

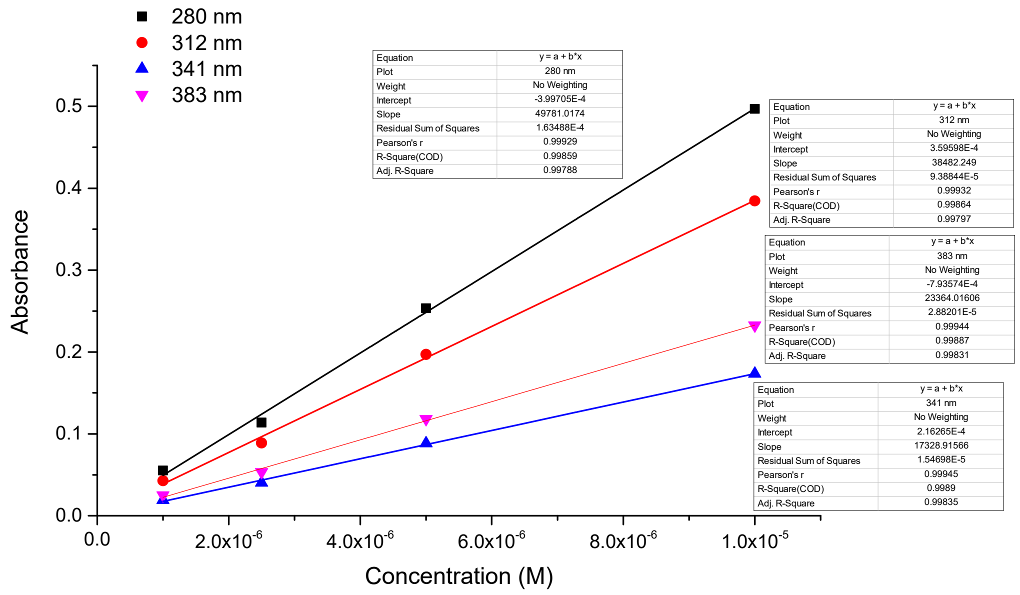

Figure S1. Absorbance vs Concentration for **[PtL^{2}Cl]**

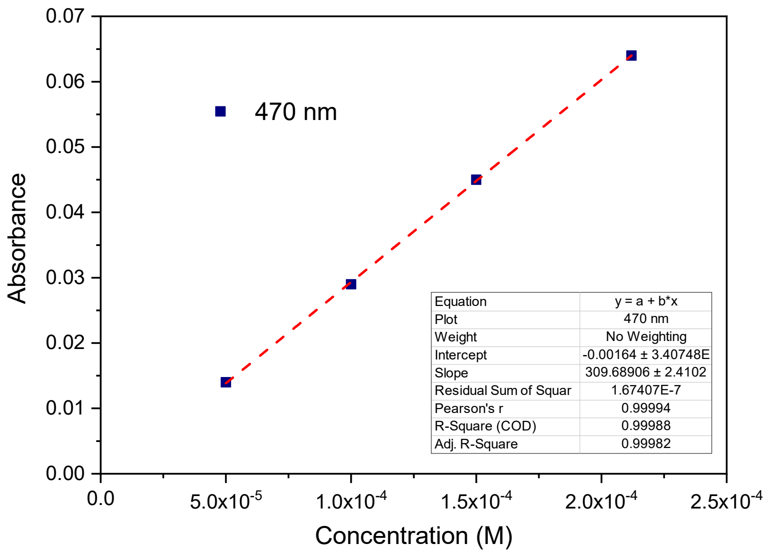

Figure S2. Absorbance vs Concentration for **[PtL^{2}Cl]** at 470 nm.

Excitation spectra

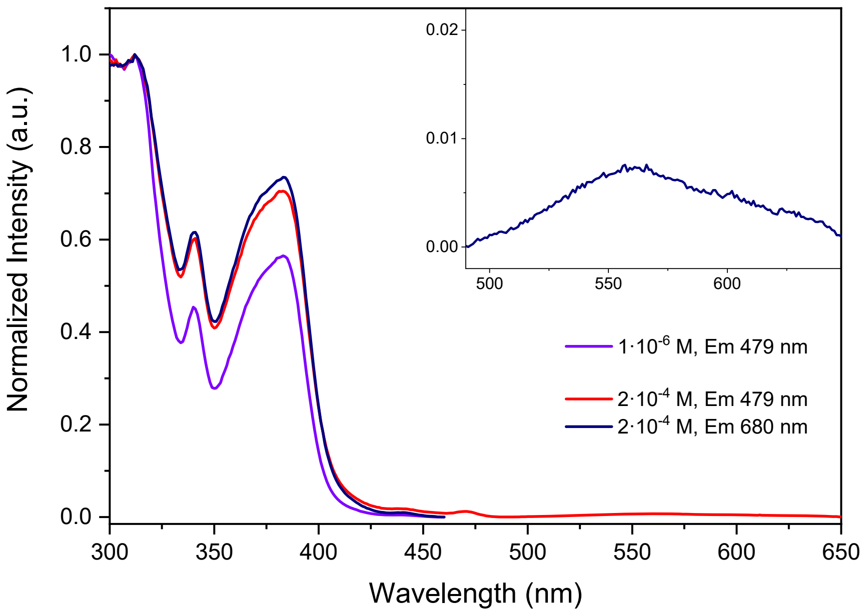

Figure S3. Excitation spectra of **[PtL²Cl]** at room temperature in dichloromethane solution at different concentrations.

Lifetime measurements

Table S2. Lifetimes of **[PtL²Cl]** at different concentrations in CH_2Cl_2; λ_{ex} = 374 nm, λ_{em} = 480 nm.

**C**	$1\cdot10^{-6}$ M	$5\cdot10^{-6}$ M	$1\cdot10^{-5}$ M	$2.1\cdot10^{-4}$ M
**τ / μs**	4.09	3.91	3.68	1.17

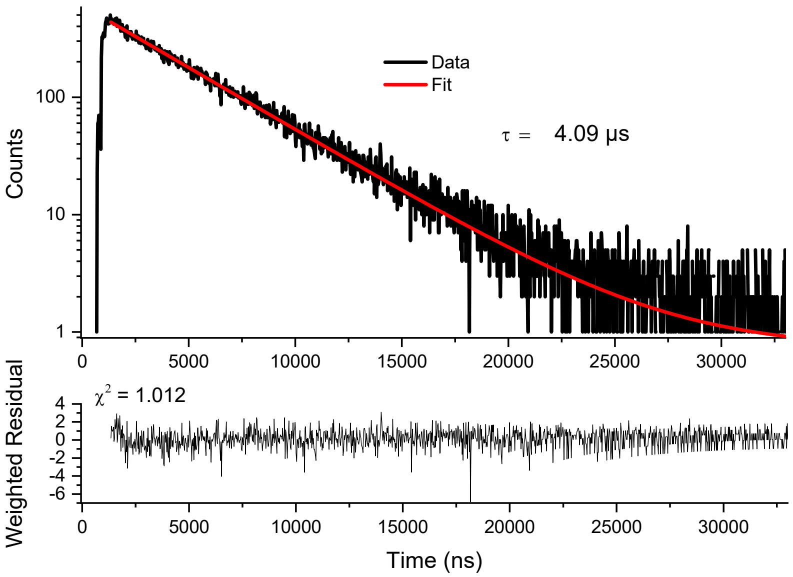

Figure S4. Lifetime measurement of **[PtL^{2}Cl]** at room temperature in dichloromethane solution ($1.0 \cdot 10^{-6}$ M); excitation 374 nm, emission 480 nm.

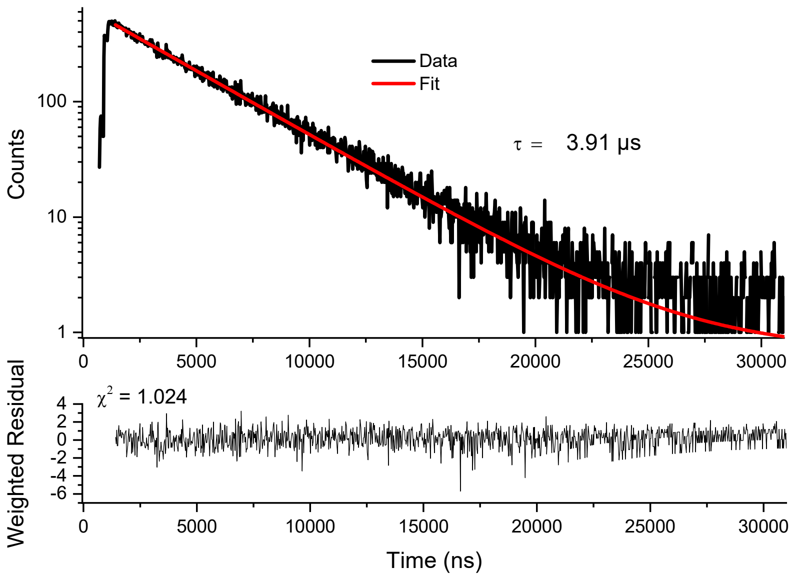

Figure S5. Lifetime measurement of **[PtL^{2}Cl]** at room temperature in dichloromethane solution ($5.0 \cdot 10^{-6}$ M); excitation 374 nm, emission 480 nm.

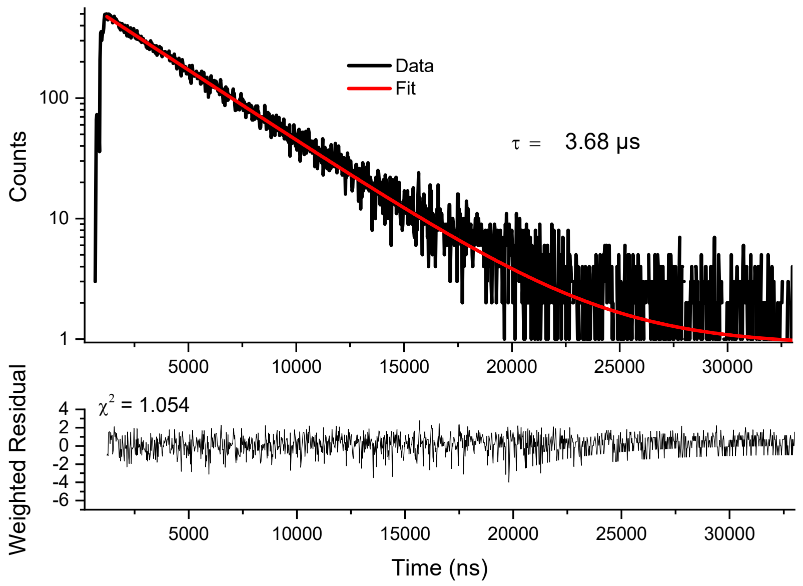

Figure S6. Lifetime measurement of **[PtL^{2}Cl]** at room temperature in dichloromethane solution ($1.0 \cdot 10^{-5}$ M); excitation 374 nm, emission 480 nm.

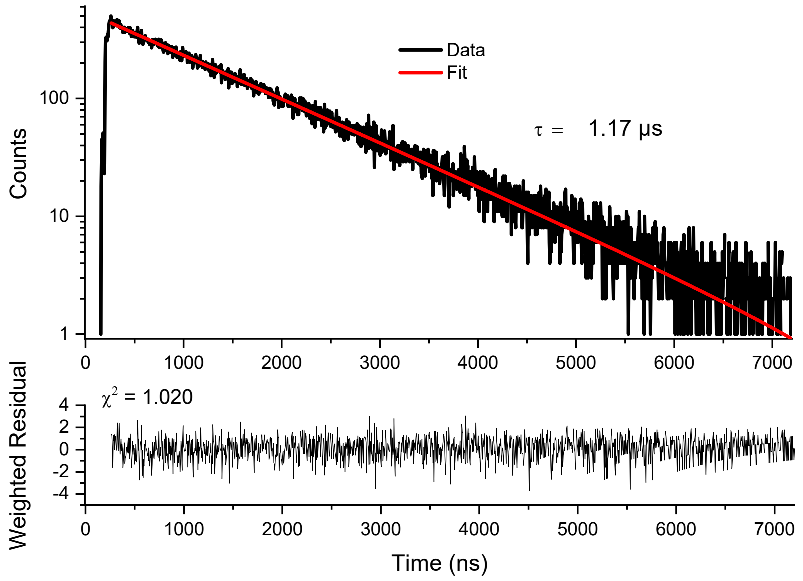

Figure S7. Lifetime measurement of **[PtL^{2}Cl]** at room temperature in dichloromethane solution ($2 \cdot 10^{-4}$ M); excitation 374 nm, emission 480 nm.

Photophysical characterization of [PtL³Cl]

UV-Vis absorption

Table S3. Molar extinction coefficients for **[PtL³Cl]** at different wavelengths.

**λ**	278 nm	311 nm	340 nm	382 nm	470 nm
**ε / M^{-1} cm^{-1}**	35134	24329	11595	15539	225

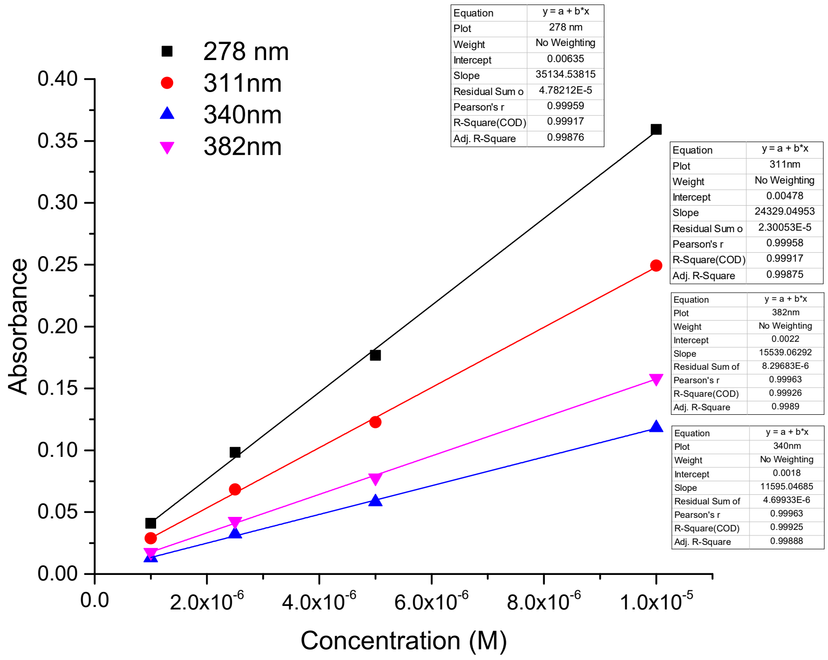

Figure S8. Absorbance vs Concentration for **[PtL³Cl]**

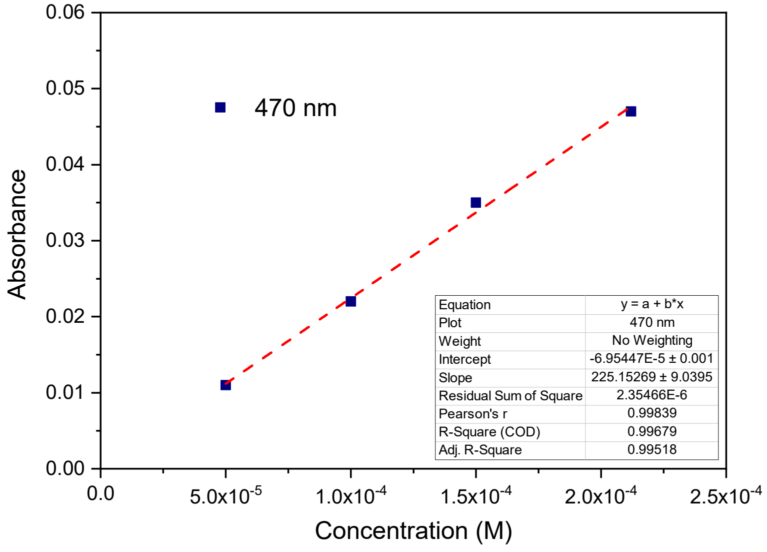

Figure S9. Absorbance vs Concentration for **[PtL³Cl]** at 470 nm.

Excitation spectra

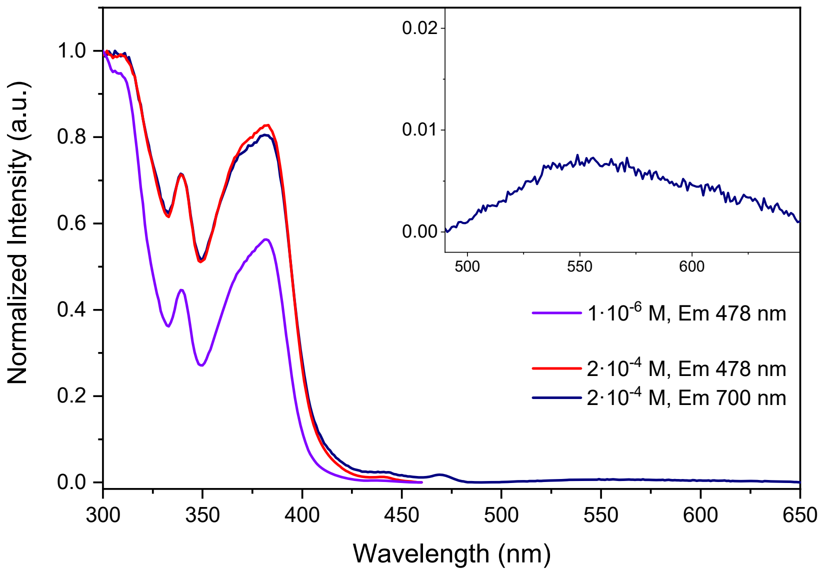

Figure S10. Excitation spectra of **[PtL³Cl]** at room temperature in dichloromethane solution at different concentrations.

Lifetime measurements

Table S4. Lifetimes of **[PtL³Cl]** at different concentrations in CH_2Cl_2; λ_{ex} = 374 nm, λ_{em} = 478 nm.

**c**	$1\cdot10^{-6}$ M	$5\cdot10^{-6}$ M	$1\cdot10^{-5}$ M	$2.1\cdot10^{-4}$ M
**τ / µs**	4.06	3.96	3.92	2.11

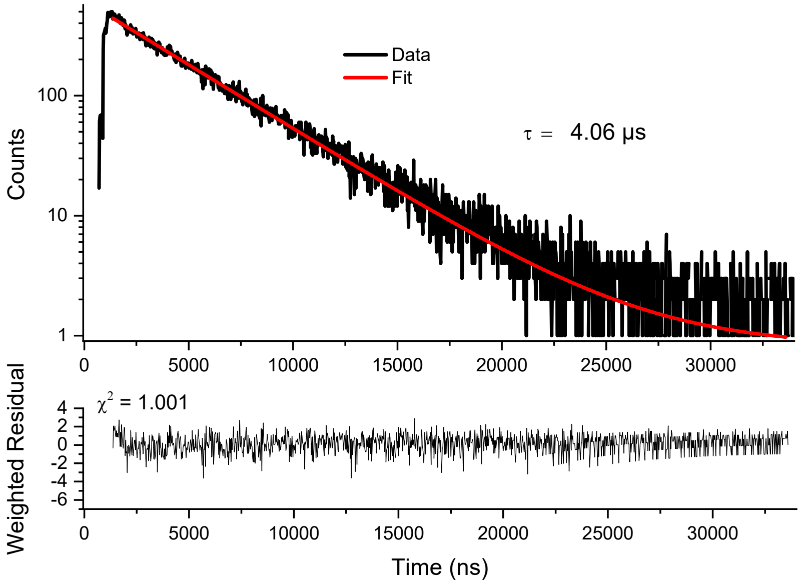

Figure S11. Lifetime measurement of **[PtL³Cl]** at room temperature in dichloromethane solution ($1\cdot10^{-6}$ M); excitation 374 nm, emission 478 nm.

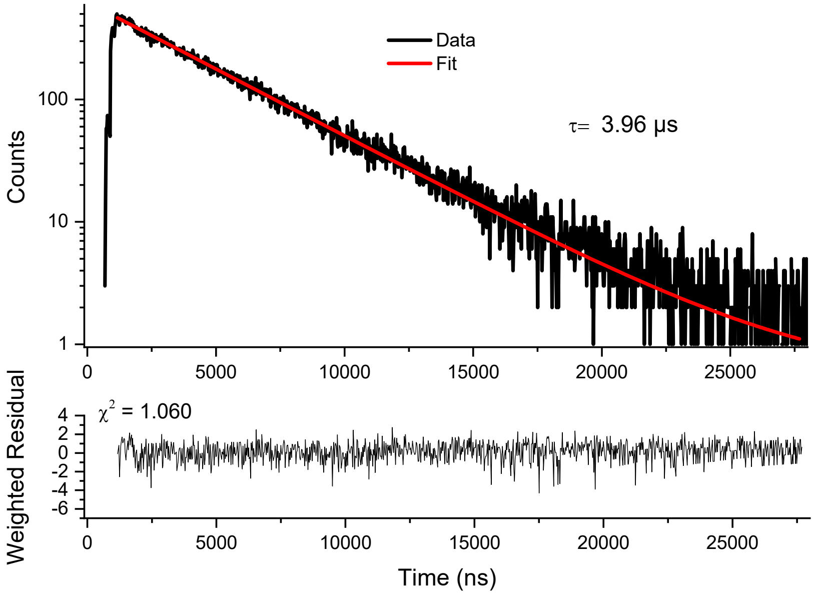

Figure S12. Lifetime measurement of **[PtL³Cl]** at room temperature in dichloromethane solution ($5\cdot10^{-6}$ M); excitation 374 nm, emission 478 nm.

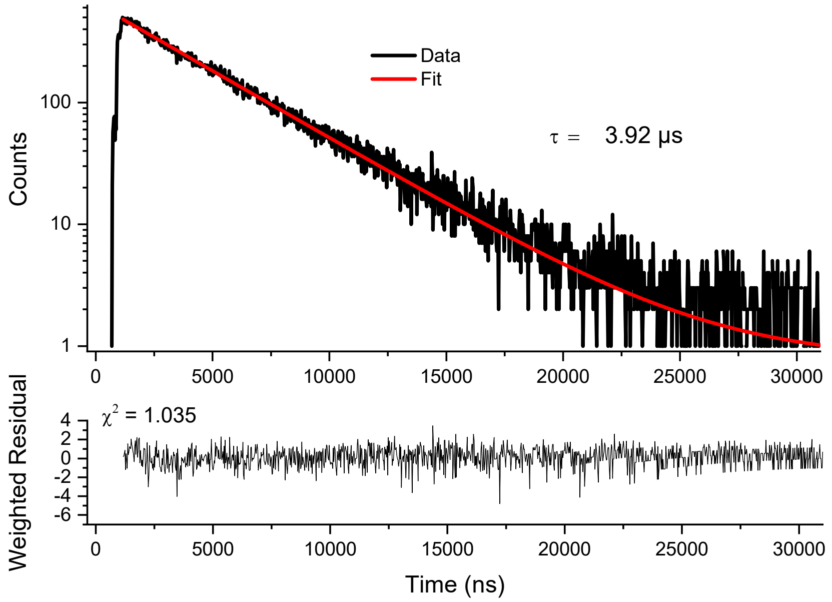

Figure S13. Lifetime measurement of **[PtL^{3}Cl]** at room temperature in dichloromethane solution ($1 \cdot 10^{-5}$ M); excitation 374 nm, emission 478 nm.

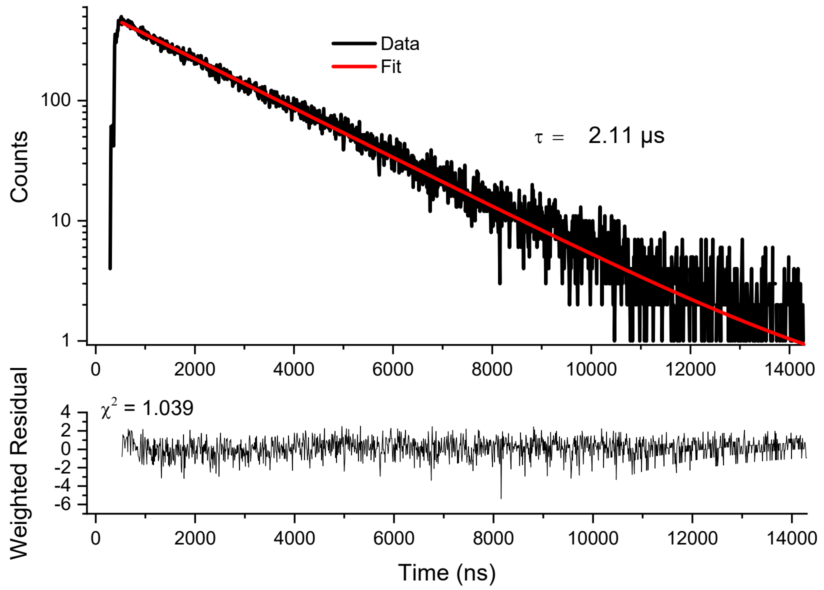

Figure S14. Lifetime measurement of **[PtL^{3}Cl]** at room temperature in dichloromethane solution ($2 \cdot 10^{-4}$ M); excitation 374 nm, emission 478 nm.

References

81. Suzuki, K.; Kobayashi, A.; Kaneko, S.; Takehira, K.; Yoshihara, T.; Ishida, H.; Shiina, Y.; Oishic, S.; Tobita, S. *Phys. Chem. Chem. Phys.*, 2009, 11, 9850–9860.
